# SPION‐mediated miR‐141 promotes the differentiation of HuAESCs into dopaminergic neuron‐like cells *via* suppressing lncRNA‐HOTAIR

**DOI:** 10.1111/jcmm.13512

**Published:** 2018-02-07

**Authors:** Te Liu, Hu Zhang, Jiajia Zheng, Jiajia Lin, Yongyi Huang, Jiulin Chen, Zhihua Yu, Lihe Guo, Weidong Pan, Ying Xiong, Chuan Chen

**Affiliations:** ^1^ Shanghai Geriatric Institute of Chinese Medicine Longhua Hospital Shanghai University of Traditional Chinese Medicine Shanghai China; ^2^ Department of Pathology Yale University School of Medicine New Haven CT USA; ^3^ Shanghai Topbiox Co Ltd Shanghai China; ^4^ Institute of Biochemistry and Cell Biology Shanghai Institutes for Biological Sciences Chinese Academy of Sciences Shanghai China; ^5^ Department of Neurology Shuguang Hospital affiliated to Shanghai University of Traditional Chinese Medicine Shanghai China; ^6^ Department of Gynaecology and Obstetrics Xinhua hospital affiliated to Shanghai Jiaotong University School of Medicine Shanghai China

**Keywords:** brain‐derived neurotrophic factor, dopaminergic neuron‐like cells (iDNLCs), human amniotic epithelial stem cells (HuAESCs), long non‐coding RNA HOTAIR, magnetofection based on superparamagnetic iron oxide nanoparticles (SPIONs), microRNA‐141 (miR‐141)

## Abstract

In this study, a bioinformatics analysis and luciferase reporter assay revealed that microRNA‐141 could silence the expression of lncRNA‐HOTAIR by binding to specific sites on lncRNA‐HOTAIR. We used superparamagnetic iron oxide nanoparticles (SPIONs) to mediate the high expression of microRNA‐141 (SPIONs@miR‐141) in human amniotic epithelial stem cells (HuAESCs), which was followed by the induction of the differentiation of HuAESCs into dopaminergic neuron‐like cells (iDNLCs). qPCR, western blot, immunofluorescence staining and HPLC all suggested that SPION‐mediated overexpression of miR‐141 could promote an increased expression of brain‐derived neurotrophic factor (BDNF), DAT and 5‐TH in HuAESC‐derived iDNLCs. The RIP and ChIP assay also showed that overexpression of miR‐141 could significantly inhibit the recruitment and binding of lncRNA‐HOTAIR to EZH2 on BDNF gene promoter. cDNA microarray analysis revealed that the expression levels of 190 genes were much higher in iDNLCs than in HuAESCs. Finally, a protein interaction network analysis and identification showed that in the iDNLC group with SPIONs@miR‐141, factors that interact with BDNF, such as FGF8, SHH, NTRK3 and CREB1, all showed significantly higher expression levels compared with those in the SPIONs@miR‐Mut. Therefore, this study confirmed that the highly efficient expression of microRNA‐141 mediated by SPIONs could improve the efficiency of HuAESCs differentiation into dopaminergic neuron‐like cells.

## Introduction

HuAESCs are derived from foetal amniotic trophoblast cells 1, 2, 3, 4. In addition to epithelial cell characteristics, these cells also have stem cell characteristics 2, 4. HuAESCs can secrete a variety of cellular growth factors, such as LIF, EGF, bFGF, TGF‐α/β and BMP‐4, and can express a variety of stem cell markers such as Nanog, Oct4, Sox2 and Nestin 2. We have previously found that HuAESCs can act as trophoblast cells to maintain the growth of embryonic stem cells and spermatogonial stem cells *in vitro* and can also be induced to differentiate into multiple types of adult cells, such as islet β‐like cells and neurons 2, 4. In addition, HuAESCs transplanted in animal models can play a role in tissue repair and regeneration 2, 5. These results indicate that HuAESCs have pluripotent differentiation potential.

LncRNA‐HOTAIR was previously investigated in the field of long non‐coding RNA. Many studies have found that lncRNA‐HOTAIR regulates the transcriptional activity of target genes *via* the recruitment of histone (de)methylation enzymes 5, 6, 7, 8, 9. LncRNA‐HOTAIR binds to the histone 3 lysine 27 (H3K27)‐specific methyltransferase complex through a motif at its 3′‐end. It also functions through a motif at its 5′‐end, which allows it to bind to the polycomb‐repressive complex 2 (PRC2, an H3K27 methylase complex, containing EZH2, SUZ12 and EED); HOTAIR and PRC2 then function together to inhibit the transcriptional activity of target genes 5, 6, 7, 8, 9. Furthermore, lncRNA‐HOTAIR functions as a scaffold in the recruitment of PRC2 and the histone demethylase complex LSD1/REST (CoREST), which catalyses H3K27 methylation and H3K4 demethylation; this in turn leads to chromatin remodelling and transcriptional inactivation of HOXD clusters and many other target genes 5, 6, 7, 8, 9. When the expression of lncRNA‐HOTAIR is inhibited by siRNA, the transcriptional activity of some target genes, such as BDNF, can be significantly up‐regulated. The expression of BDNF mRNA is also greatly increased, which suggests that lncRNA‐HOTAIR negatively regulates the transcription and expression of BDNF 5.

Magnetic nanomaterials are a type of nanomaterial that have a particle size between 0 and 100 nm. They generally consist of iron, cobalt, nickel and their alloys, which can directly or indirectly produce magnetism. Among a diverse array of magnetic nanoparticles, oxide nanoparticles have been widely investigated in recent years because of their high magnetic saturation strength, low toxicity, easy availability of raw materials and high surface reactivity. In recent years, oxide nanoparticles have also received much attention for their application as gene carriers. When their size is less than 20 mm, magnetic nanoparticles often exhibit superparamagnetism. SPIONs possess controllable features and good stability and can be easily modified. They have thus become the current focus of gene carrier research. After they bind with plasmid DNA and siRNA, SPIONs can transfer the nucleic acids into mammalian cells in the presence of an external magnetic field. The local DNA concentration can be increased when the internal and external barriers of cells are overcome through magnetic adsorption, which improves the transfection efficiency 7, 10, 11, 12. Further studies have found that surface modification of SPIONs using cationic liposomes or cationic polymers, such as polyethyleneimine (PEI), dendrimers (PAMAM, PPI), dextran and chitosan, can facilitate additional interactions between the nanomaterials and the nucleic acids to be transfected, which has contributed to the improvement in transfection efficiency 7, 10, 11, 12. Our previous studies have confirmed that SPIONs can effectively bind to microRNAs or siRNAs, mediate their expression in cells and inhibit tumour cell proliferation and invasion 7, 10.

However, the regulatory mechanism by which lncRNA‐HOTAIR induces the differentiation of HuAESCs into iDNLCs *in vitro* is not clear. Therefore, this study focused on the microRNA‐lncRNA‐BDNF axis and investigated in great detail the function of microRNA‐mediated regulation of lncRNA‐HOTAIR expression in the induced differentiation of HuAESCs into iDNLCs. Our results showed that SPION‐mediated overexpression of exogenous microRNA‐141 in HuAESCs could effectively inhibit the expression of endogenous lncRNA‐HOTAIR, increase the expression of BDNF and, finally, promote the differentiation of HuAESCs into iDNLCs.

## Material and methods

### Separation and culture of HuAESCs

The separation and culture of HuAESCs were performed as described in our previous publications 1, 3, 4. Briefly, human amniotic membrane was collected from the First Maternal and Child Health Centre of Shanghai, China. The amniotic membrane was then minced and digested in 0.25% trypsin (containing 0.02% EDTA) to isolate epithelial stem cells. HuAESCs were grown in DMEM:F12 (1:1) cell culture medium supplemented with 5% foetal bovine serum, 10% KnockOut™ Serum Replacement, 10 ng/ml basic fibroblast growth factor (bFGF) and 10 ng/ml epidermal growth factor (EGF) and were cultured at 37°C in 5% CO_2_ for 72 hr.

### Induced differentiation of DNLCs

Briefly, second‐generation HuAESCs were digested in 0.25% trypsin and seeded into a 6‐well cell culture plate. After the cells became completely adherent the following day, the medium was replaced with DMEM:F12 (1:1) cell culture medium supplemented with 50 ng/ml FGF8a, 100 ng/ml SHH, N2 plus B27 supplements, 200 IM ascorbic acid, 20 ng/ml BDNF and 20 ng/ml glial cell‐derived neurotrophic factor (GDNF). The cells were maintained in culture for 5 weeks 13, 14.

### Induction of the transfection of microRNA into cells using SPIONs

SPIONs were purchased from Novobio (Novobio Biotechnology Co., Ltd, Shanghai, China). According to the manufacturer's instructions and previously published methods, 5 μl of 0.2 mM SPIONs was thoroughly mixed with 5 μl of 10 μM miR‐141 oligonucleotide RNA(UAACACUGUCUGGUAAAGAUGG) (Sigma‐Aldrich, St. Louis, USA) or miR‐Mut(UcACtCaGcCUcGUgAucAUuG), vortexed for 10 s and then maintained at room temperature for 20 min. A total of 10 μl of the SPION‐miR mixture was then combined with 90 μl of DMEM:F12 (1:1) serum‐free medium, added to 1 × 10^4^ cells/ml and incubated for 72 hr at 37°C in 5% CO_2_.

### RNA extraction and analysis by quantitative real‐time PCR (qPCR)

Total RNA was extracted from each group of cells using TRIzol reagent according to the manufacturer's instructions (Invitrogen). Total RNA was treated with DNase I (Sigma‐Aldrich), quantified and subjected to reverse transcription using a ReverTra Ace‐α First Strand cDNA Synthesis Kit (Toyobo (Shanghai) Biotech Co., Ltd., Shanghai, China) to generate cDNA. qRT‐PCR was performed in a RealPlex 4 real‐time PCR detection system (Eppendorf Co. Ltd, Shanghai, China). SyBR Green RealTime PCR Master Mix (Toyobo) was used as the fluorescent dye for nucleic acid amplification. The qRT‐PCR included a total of 40 amplification cycles of denaturation at 95°C for 15 s, annealing at 58°C for 30 sec. and extension at 72°C for 42 sec. The relative gene expression level was determined by the 2‐ΔΔCt method, wherein ΔCt=Ct_genes‐Ct_18sRNA and ΔΔCt= ΔCt_all_groups‐ΔCt_blankcontrol_group. The expression level of the mRNA was normalized to the expression level of 18s rRNA.

### Transmission electron microscopy (TEM) analysis

The samples were fixed and embedded according to procedures described previously 7, 10. Tissue samples were first fixed in 2.5% glutaraldehyde (Sigma‐Aldrich) for 4 hand then fixed in 1% osmium tetroxide for 1 hr, followed by dehydration in acetone; finally, the samples were embedded in resin 12 (Ted Pella, Inc., CA, USA). Ultrathin slices (thickness of 70 nm) of the samples were generated and were attached to a copper mesh. The sections were stained with 1% uranium acetate (Sigma‐Aldrich) and 1% lead citrate (Sigma‐Aldrich) and were then imaged with a JEM‐1230 transmission electron microscope (JEOL Ltd., Tokyo, Japan).

### Immunofluorescence analysis

Briefly, the cells were fixed in 4% paraformaldehyde (Sigma‐Aldrich) for 15 min. and then blocked in a blocking solution (Beyotime Biotechnology Co., Ltd, Zhejiang, China) at 37°C for 30 min. The blocking solution was discarded, and the cells were washed three times in wash buffer at room temperature for 1 min. each time. Subsequently, the cells were incubated with the primary antibody (Table S1) at 37°C for 45 min. The antibody was then discarded, and the cells were washed three times in wash buffer at room temperature for 5 min. each time. The secondary antibody (Table S1) was then added, followed by incubation at 37°C for 45 min. The antibody was then discarded, and cells were washed three times in wash buffer at room temperature for 5 min. each time. Finally, the slides were mounted with immunofluorescence anti‐quenching mounting medium and coverslipped.

### Northern blot

Briefly, total RNA was extracted from all the cell groups using TRIzol reagent. After quantification, 20 g of high‐quality total RNA was selected to be used for 7.5 M urea‐12% formaldehyde (PAA) denaturing gel electrophoresis, followed by transfer to a Hybond N + nylon membrane (Amersham, Freiburg, Germany). The membrane was cross‐linked with 1200 mjoule/cm2 UV for 30 s. Hybridization was performed with miR‐141 and HOTAIR antisense DNA probes to detect miR‐141 expression. After hybridization and washing, the membrane was exposed to Kodak XAR‐5 film for 20–40 hr (Sigma‐Aldrich Chemical). As a positive control, all membranes were hybridized with the human U6 snRNA probe (5′‐GCAGGGGCCATGCTAATCTTCTCTGTATCG‐3′). The exposure time of the U6 snRNA probe was maintained between 15 and 30 min. 10.

### Western blot

Briefly, total protein was extracted from each group of cells and was subjected to 12% SDS‐PAGE denaturing gel electrophoresis, followed by transfer to a PVDF membrane (Millipore). After the membrane was blocked and washed, the blot was incubated with the primary antibodies at 37°C for 45 min. (Table S1). After thorough washing, the blot was incubated with the secondary antibodies at 37°C for 45 min. The membrane was washed four times in TBST at room temperature for 14 min. each time and was then subjected to exposure and development (Sigma‐Aldrich Chemical) using enhanced chemiluminescence (ECL, Pierce Biotechnology, Rockford, IL, USA).

### ChIP

Briefly, cells were fixed in 1% paraformaldehyde for 30 min. at 37°C. Then, 125 mM glycine was added, followed by incubation at room temperature for 10 min. before the cross‐linking was terminated. The sample was then subjected to ultrasonication on ice to fragment the DNA into 200–1000‐bp chromatin fragments. The primary antibodies (Table S1) were then added, which was followed by incubation overnight at 4°C. Protein A/G plus‐agarose was added to obtain immunoprecipitates, which was followed by PCR amplification. PCR amplification was performed for 33 cycles as follows: denaturation at 95°C for 30 sec., annealing at 55°C for 30 sec. and extension at 72°C for 30 sec. The amplification products were measured by agarose gel electrophoresis.

### RNA immunoprecipitation (RIP) assay

Briefly, all cells were fixed in 1% paraformaldehyde for 30 min. at 37°C. Then, 125 mM glycine was added, followed by incubation at room temperature for 10 min. before the cross‐linking was terminated. Then, the Nuclear Protein Extraction Kit (Beyotime Biotechnology, Hangzhou, China) was used to extract the nuclear proteins of all cells. The primary antibodies (Table S1) were then added, which was followed by incubation overnight at 4°C. Protein A/G plus‐agarose beads were added to obtain immunoprecipitates. Then, the coprecipitated RNAs by resuspending beads were added in TRIzol RNA extraction reagent (Invitrogen) 1 ml and were extracted using the QIAGEN RNeasy Mini Kit (QIAGEN, Shanghai, China). Total RNAs were quantified and subjected to reverse transcription using a ReverTra Ace‐α First Strand cDNA Synthesis Kit (Toyobo) to generate cDNA *in vitro*. PCR amplification was performed for 31 cycles as follows: denaturation at 95°C for 30 sec., annealing at 65°C for 30 sec. and extension at 72°C for 42 sec. The amplification products were measured by agarose gel electrophoresis.

### Microarray analysis

Briefly, total cellular RNA was extracted using the Qiagen RNeasy Mini Kit, and the cDNA first and second chains were synthesized *in vitro*. Subsequently, the fluorescent labelling of cRNA was completed using the Quick Amp Labeling Kit, One‐Color (Agilent p/n 5190‐2305). The cRNA content was measured, and the cRNA concentration was adjusted using the following formula: cRNA concentration=RNAm‐ (total RNAi) (y), where RNAm=cRNA amount measured after IVT (μg), total RNAi=total initial RNA amount (μg) and y=percentage of double‐stranded cDNA added during IVT out of the total cDNA product. The Cy3‐tagged cRNA was hybridized to the Agilent Human Gene Expression gene chip (8 * 60K, Design ID: 039494) (Oebiotech, Shanghai, China). After the reaction, the chips were scanned using an Agilent scanner with a resolution of 5 μm. The scanner automatically scanned once each time at 100% and 10% PMT, and the two results were automatically combined by Agilent software.

### Statistical analysis

Each experiment was performed at least three times, and data are shown as mean ± S E where applicable, and differences were evaluated using Student's *t*‐tests. The probability of *P* < 0.05 was considered to be statistically significant.

## Results

### Targeted regulation of HOTAIR expression by miR‐141

**Figure 1:**
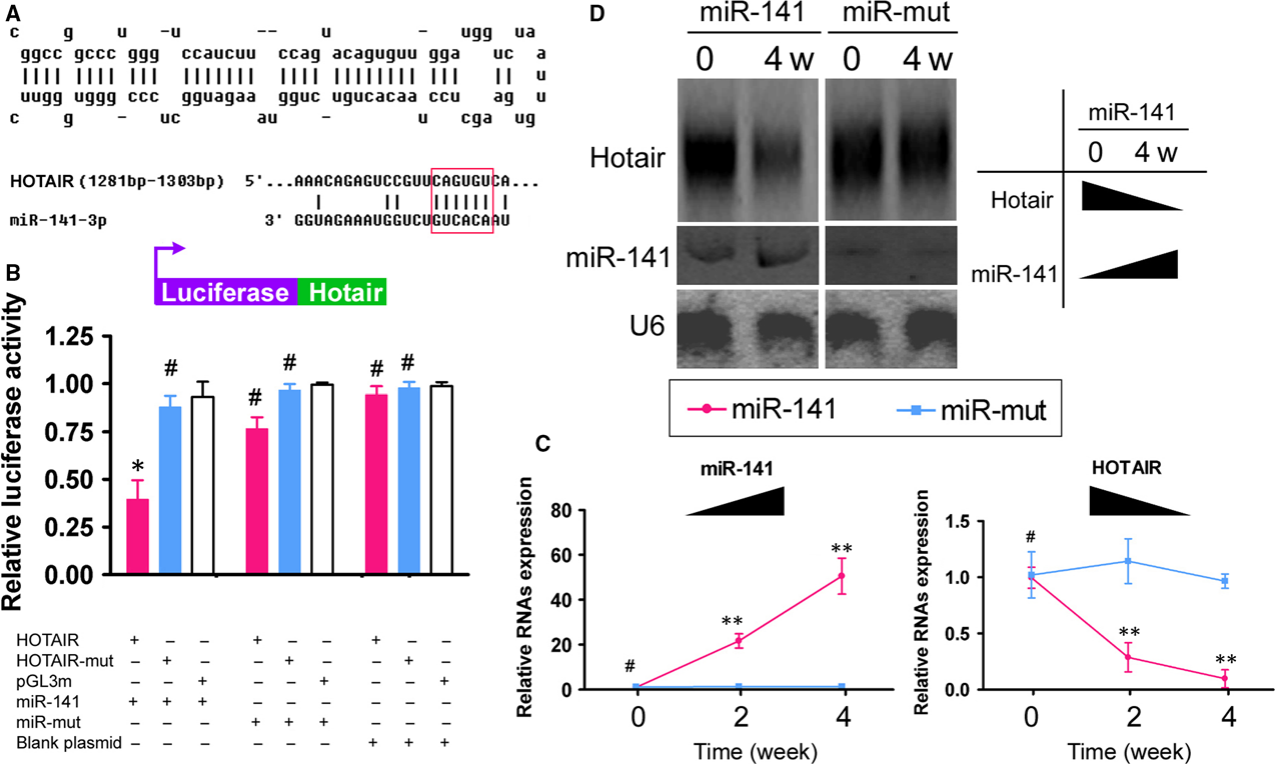
Targeted control of HOTAIR expression by miR‐141(**A**) miR‐141 secondary structure and complete complementation of miR‐141 and 6 bp located within a specific site (1295 bp–1300 bp) of lncRNA‐HOTAIR. (**B**) Luciferase assay showed that when WT miR‐141 was overexpressed in cells, the luciferase expression of constructs carrying WT HOTAIR was significantly decreased, while the remaining combinations did not affect luciferase expression; *P < 0.05 *vs*. pGL3 m, *n* = 3; ^#^ *P* > 0.05 *vs*. pGL3 m, *n* = 3. (**C**) qPCR showed that upon SPION‐mediated miR‐141 overexpression in HuAESCs, the expression of miR‐141 was significantly increased with prolonged iDNLC induction time, whereas the expression of endogenous HOTAIR was significantly decreased with prolonged induced differentiation time; **P < 0.01 *vs*. pGL3 m, *n* = 3; ^#^ *P* > 0.05 *vs*. pGL3 m, *n* = 3. (D) Northern blot results showed that the HOTAIR hybridization signal in the SPIONs@miR‐141‐iDNLCs group in the 4^th^ week was significantly weaker than that on day 0, while the hybridization signal of miR‐141 was significantly stronger in the 4^th^ week than on day 0.

The bioinformatics analysis showed that mature miR‐141 can complement and fully pair with six bases at a specific site within lncRNA‐HOTAIR (1295 bp–1300 bp), which suggests that HOTAIR may be a target of miR‐141 (Fig. 1A). A luciferase assay showed that when WT miR‐141 was overexpressed in cells, the luciferase expression of constructs carrying WT HOTAIR was significantly decreased, while the remaining combinations did not affect luciferase expression (Fig. 1B). In addition, qPCR showed that upon SPION‐mediated miR‐141 overexpression in HuAESCs, the expression of miR‐141 was significantly increased with prolonged iDNLC induction time, whereas the expression of endogenous HOTAIR was significantly decreased with prolonged induced differentiation time (Fig. 1C). Northern blot results showed that the HOTAIR hybridization signal in the SPIONs@miR‐141‐iDNLCs group in the 4th week was significantly weaker than that on day 0, while the miR‐141 hybridization signal was significantly stronger in the 4^th^ week than on day 0 (Fig. 1D). However, in the SPIONs@miR‐Mut‐iDNLCs group, no significant differences were observed in the intensity of the hybridization signals of either HOTAIR or miR‐141 at any of the time points (Fig. 1D). These results indicate that lncRNA‐HOTAIR is one of the specific targets of miR‐141 and that overexpression of miR‐141 inhibits the expression of endogenous HOTAIR in HuAESCs during induction.

### SPIONs mediated the expression of exogenous miR‐141 in HuAESCs and promoted the expression of dopaminergic neuron markers

**Figure 2:**
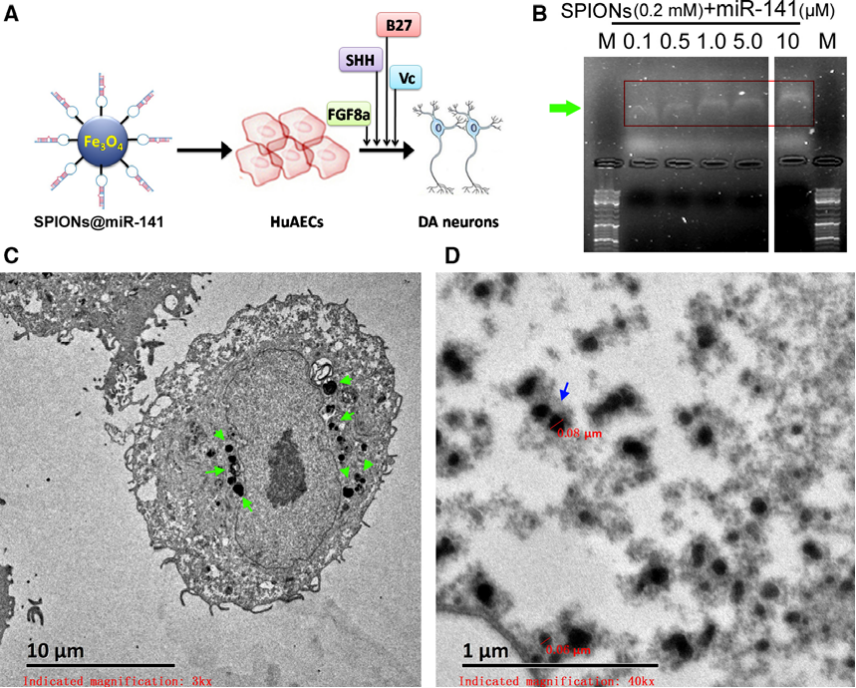
SPIONs and miR‐141 cross‐linking *in vitro* (**A**) SPION‐mediated miR‐141 expression in HuAESCs promoted their targeted differentiation into dopaminergic neurons. (**B**) Gel imaging results show that different concentrations (0.1 μM‐10 μM) of miR‐141 oligonucleotides can be successfully cross‐linked with SPIONs (0.2 mM) *in vitro*; M is a DNA marker. The arrows indicated different concentrations of miR‐141. (**C**) and (**D**) Transmission electron microscopy showed that large quantities of SPIONs were aggregated in HuAESCs and formed dense electron clouds with a particle size of approximately 80 nm. The arrows indicated SPIONs.

**Figure 3:**
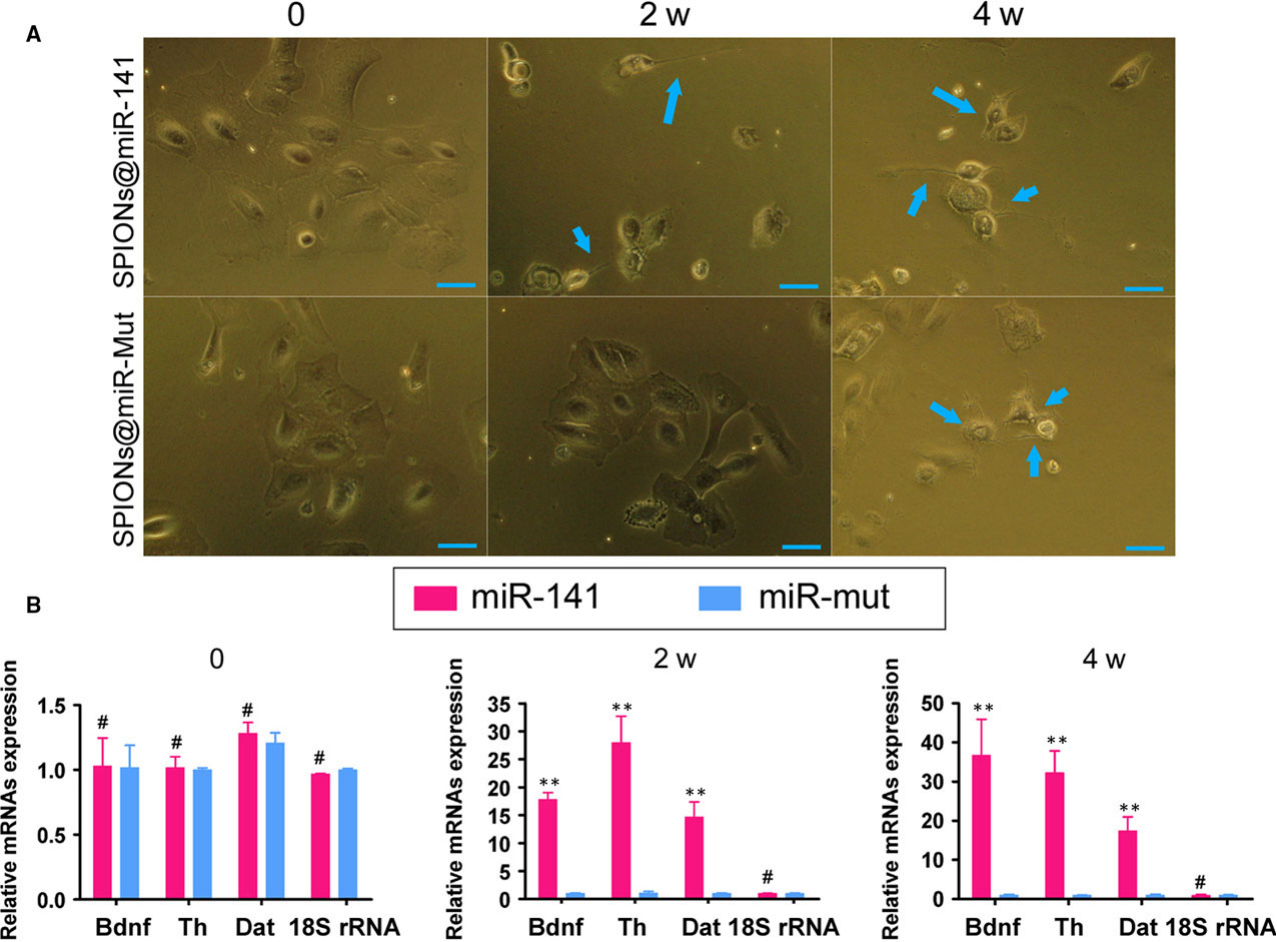
SPIONs@miR‐141 facilitated the differentiation of HuAESCs into iDNLCs (**A**) Difference in the induced differentiation of SPIONs@miR‐141‐HuAESCs and SPIONs@miR‐Mut‐HuAESCs into iDNLCs by light microscopy. During the 4^th^ week of induction, although the SPIONs@miR‐Mut‐HuAESCs group also contained small numbers of iDNLCs, the number of iDNLCs in this group was much lower than that in the SPIONs@miR‐141‐HuAESCs group. (**B**) qPCR results showed that during the 2^nd^ and 4^th^ weeks of induction, the mRNA expression levels of the dopaminergic neuron markers TH and DAT, as well as the level of BDNF, in iDNLCs derived from SPIONs@miR‐141‐HuAESCs were higher than those in iDNLCs derived from SPIONs@miR‐Mut‐HuAESCs; **P < 0.01 *vs*. SPIONs@miR‐Mut‐HuAESCs, *n* = 3; ^#^ *P* > 0.05 *vs*. SPIONs@miR‐Mut‐HuAESCs, *n* = 3.

**Figure 4:**
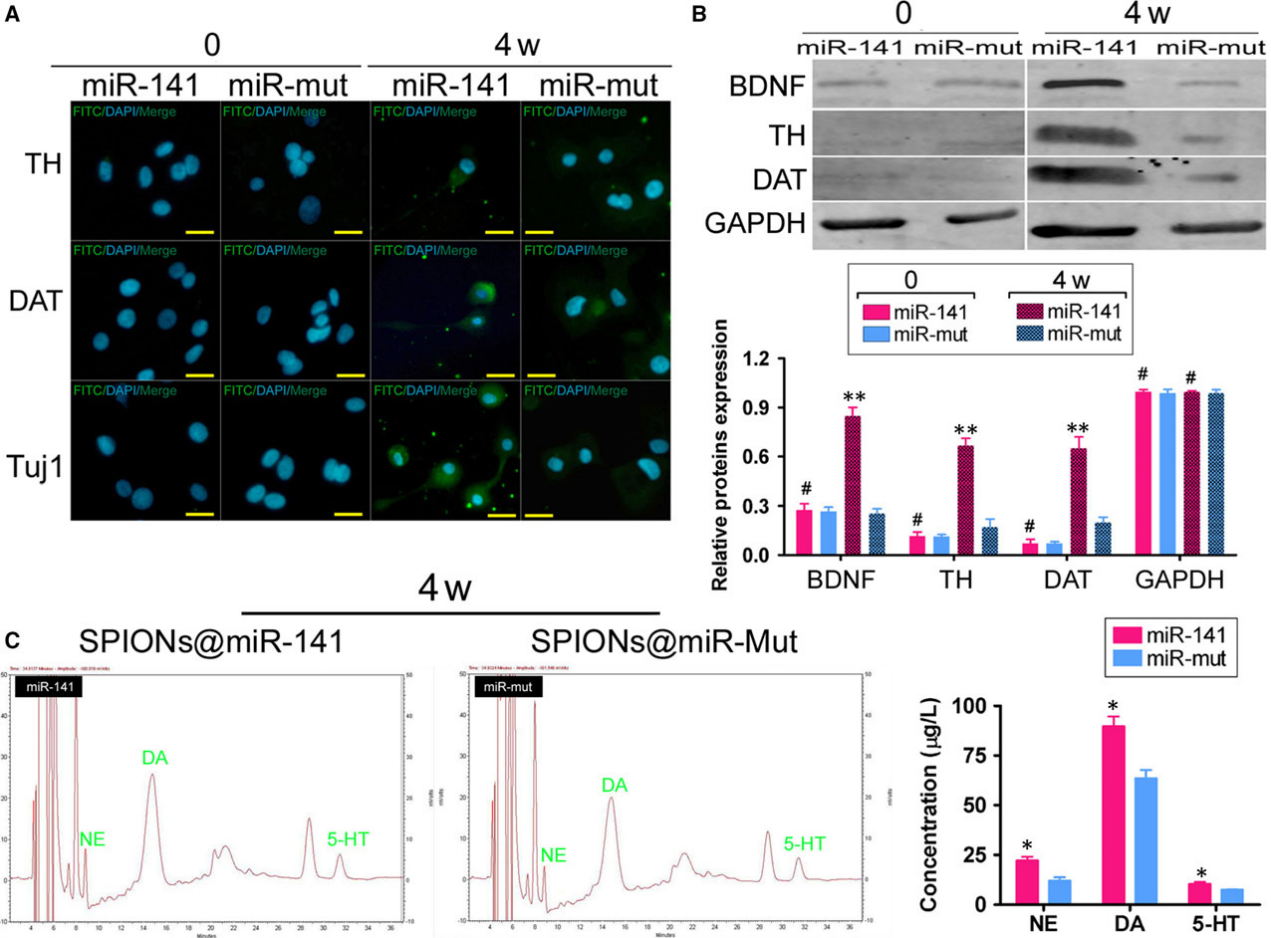
SPIONs@miR‐141 promoted the expression of dopaminergic neuron markers in HuAESCs (**A**) Immunofluorescence staining showed that iDNLCs derived from SPIONs@miR‐141‐HuAESCs had high expression levels of BDNF, TH, DAT and TUJ1 proteins. (**B**) Western blot results showed that iDNLCs derived from SPIONs@miR‐141‐HuAESCs had high expression levels of BDNF, TH and DAT proteins; **P < 0.01 *vs*. SPIONs@miR‐Mut‐HuAESCs, *n* = 3; ^#^ *P* > 0.05 *vs*. SPIONs@miR‐Mut‐HuAESCs, *n* = 3. (**C**) HPLC also suggested that the level of DA secreted by iDNLCs derived from SPIONs@miR‐141‐HuAESCs was significantly higher than that secreted by iDNLCs derived from SPIONs@miR‐Mut‐HuAESCs;*P < 0.05 *vs*. SPIONs@miR‐Mut‐HuAESCs, *n* = 3; ^#^ *P* > 0.05 *vs*. SPIONs@miR‐Mut‐HuAESCs, *n* = 3.

We first cross‐linked different concentrations of miR‐141 oligonucleotides with SPIONs *in vitro* and then analysed the cross‐linking conditions by agarose gel electrophoresis. The results of gel imaging showed that the different concentrations (0.1 μM–10 μM) of miR‐141 oligonucleotides could all be successfully cross‐linked with SPIONs (0.2 mM) *in vitro* (Fig. 2B). Therefore, miR‐141 was used at a concentration of 10 μMin. subsequent experiments. After 48 hr of coincubation of HuAESCs with SPIONs@miR‐141 *in vitro*, transmission electron microscopy revealed multiple dense electron clouds with a diameter between 60 and 80 nm in the cytoplasm, which were presumably SPIONs (Fig. 2C and D). Subsequently, SPIONs@miR‐141 and SPIONs@miR‐Mut were each cocultured with HuAESCs, which was followed by the induction of HuAESC differentiation into iDNLCs. At first, no significant difference was observed in the morphology between the two groups, both of which comprised epithelioid cells. During the second week of induction, a small number of neuron‐like cells appeared in the SPIONs@miR‐141‐HuAESCs group; these cells had oval‐shaped soma and unipolar or bipolar synaptic‐like pseudopods (Fig. 3A). In contrast, cells in the SPIONs@miR‐Mut‐HuAESCs group did not have the phenotype described above. In the 4^th^ week of induction, although the SPIONs@miR‐Mut‐HuAESCs group also contained small numbers of iDNLCs, the number of iDNLCs in this group was much smaller than that in the SPIONs@miR‐141‐HuAESCs group (Fig. 3A). qPCR showed that in the 2^nd^ and 4^th^ weeks of induction, the mRNA expression levels of the dopaminergic neuron markers TH and DAT, as well as the levels of BDNF, in iDNLCs derived from SPIONs@miR‐141‐HuAESCs were higher than those in iDNLCs derived from SPIONs@miR‐Mut‐HuAESCs (Fig. 3B). Immunofluorescence staining and western blot both demonstrated that iDNLCs derived from SPIONs@miR‐141‐HuAESCs had high expression levels of BDNF, TH, DAT and TUJ1 proteins (Fig. 4A and B). In addition, HPLC results also suggested that although cells in both groups could secrete dopamine (DA), norepinephrine (NE) and serotonin (5‐HT) during the 4^th^ week of induction, the level of DA secreted by iDNLCs derived from SPIONs@miR‐141‐HuAESCs was significantly higher than that secreted by iDNLCs derived from SPIONs@miR‐Mut‐HuAESCs (Fig. 4C). The results indicate that SPION‐mediated exogenous miR‐141 expression in HuAESCs can promote the differentiation of HuAESCs into iDNLCs.

### miR‐141 inhibited endogenous lncRNA‐HOTAIR recruitment of transcriptional repressors

**Figure 5:**
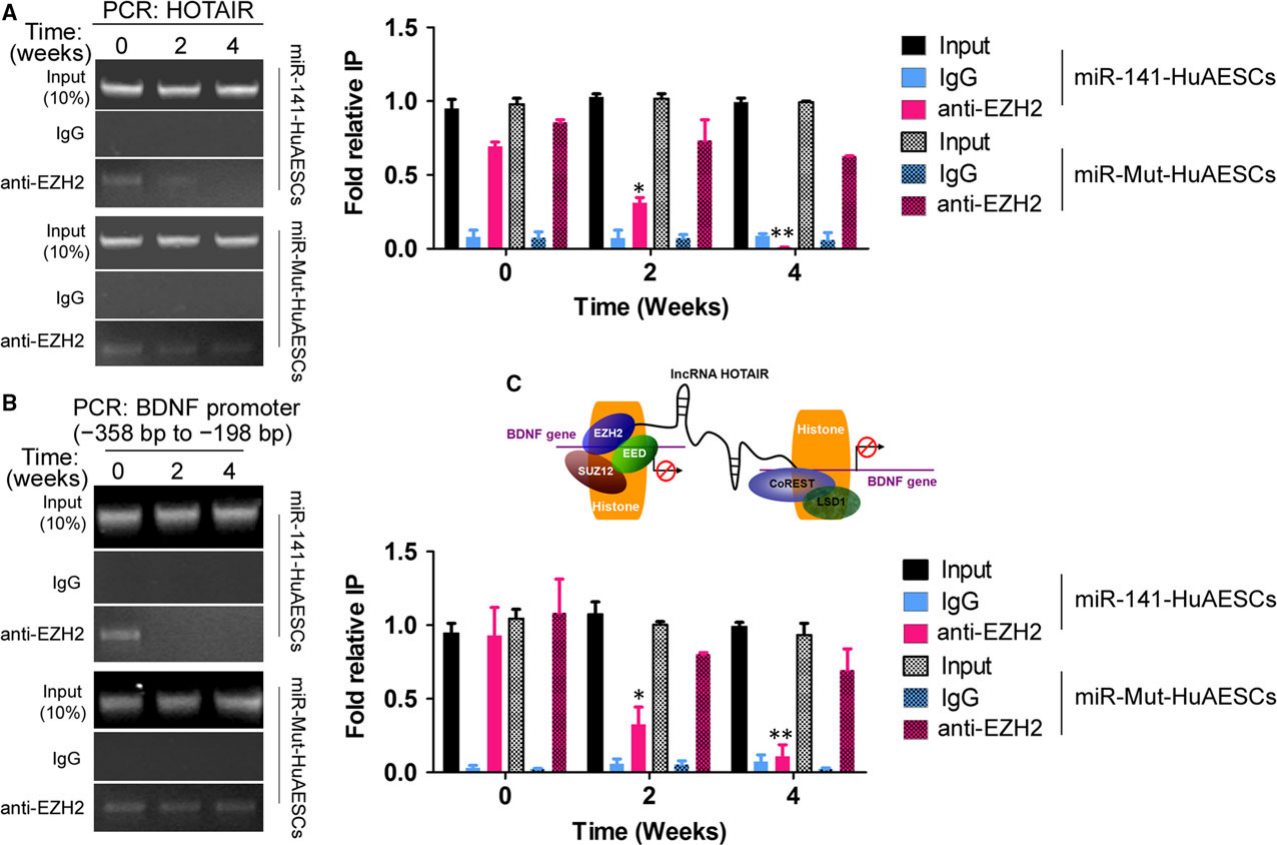
Inhibition of endogenous lncRNA‐HOTAIR recruitment of transcriptional repressors by miR‐141 (**A**) RIP results suggested that in iDNLCs derived from SPIONs@miR‐141‐HuAESCs, the level of HOTAIR bound to EZH2 in the nucleus was significantly decreased as the induction time increased and was significantly different compared with the level in the control group;**P < 0.01 *vs*. SPIONs@miR‐Mut‐HuAESCs, *n* = 3; *P < 0.05 *vs*. SPIONs@miR‐Mut‐HuAESCs, *n* = 3. (**B)** The specified region in the BDNF promoter (−358 bp to −198 bp) was tested by ChIP‐PCR assay. ChIP results showed that in iDNLCs derived from SPIONs@miR‐141‐HuAESCs, the level of EZH2 bound to the BDNF gene promoter region was significantly decreased as the induction time increased, and this level was significantly different from that in the control group; **P < 0.01 *vs*. SPIONs@miR‐Mut‐HuAESCs, *n* = 3. (**C**) The endogenous lncRNA‐HOTAIR could recruit transcriptional inhibitor EZH2 into the nucleus and binding to the promoter of its target gene BDNF, which would suppress transcriptional activation of that target gene.

Previous studies have shown that lncRNA‐HOTAIR can recruit transcriptional inhibitory complexes that bind to specific histone sites (H3K4 and H3K27), which leads to the inhibition of gene transcription 5, 7, 9. In this study, RIP results suggested that in iDNLCs derived from SPIONs@miR‐141‐HuAESCs, the frequency at which HOTAIR bound to EZH2 in the nucleus was significantly decreased as the induction time increased, and a significant difference was seen compared with the control group (SPIONs@miR‐Mut‐HuAESCs) (Fig. 5A). The specified region in the BDNF promoter (−358 bp to −198 bp) was tested by ChIP‐PCR assay. ChIP results showed that in iDNLCs derived from SPIONs@miR‐141‐HuAESCs, the level of EZH2 bound to the BDNF gene promoter region was significantly decreased as the induction time increased, and this was significantly different compared with what was observed in the control group (SPIONs@miR‐Mut‐HuAESCs) (Fig. 5B). These results demonstrate that overexpression of miR‐141 can inhibit endogenous lncRNA‐HOTAIR recruitment of the transcriptional inhibitor EZH2. This would prevent the entry of EZH2 into the nucleus and its binding to the promoter of its target gene BDNF, which would promote transcriptional activation of that target gene (Fig. 5C).

### miR‐141 promoted the expression of BDNF and dopaminergic neuron‐associated proteins

**Figure 6:**
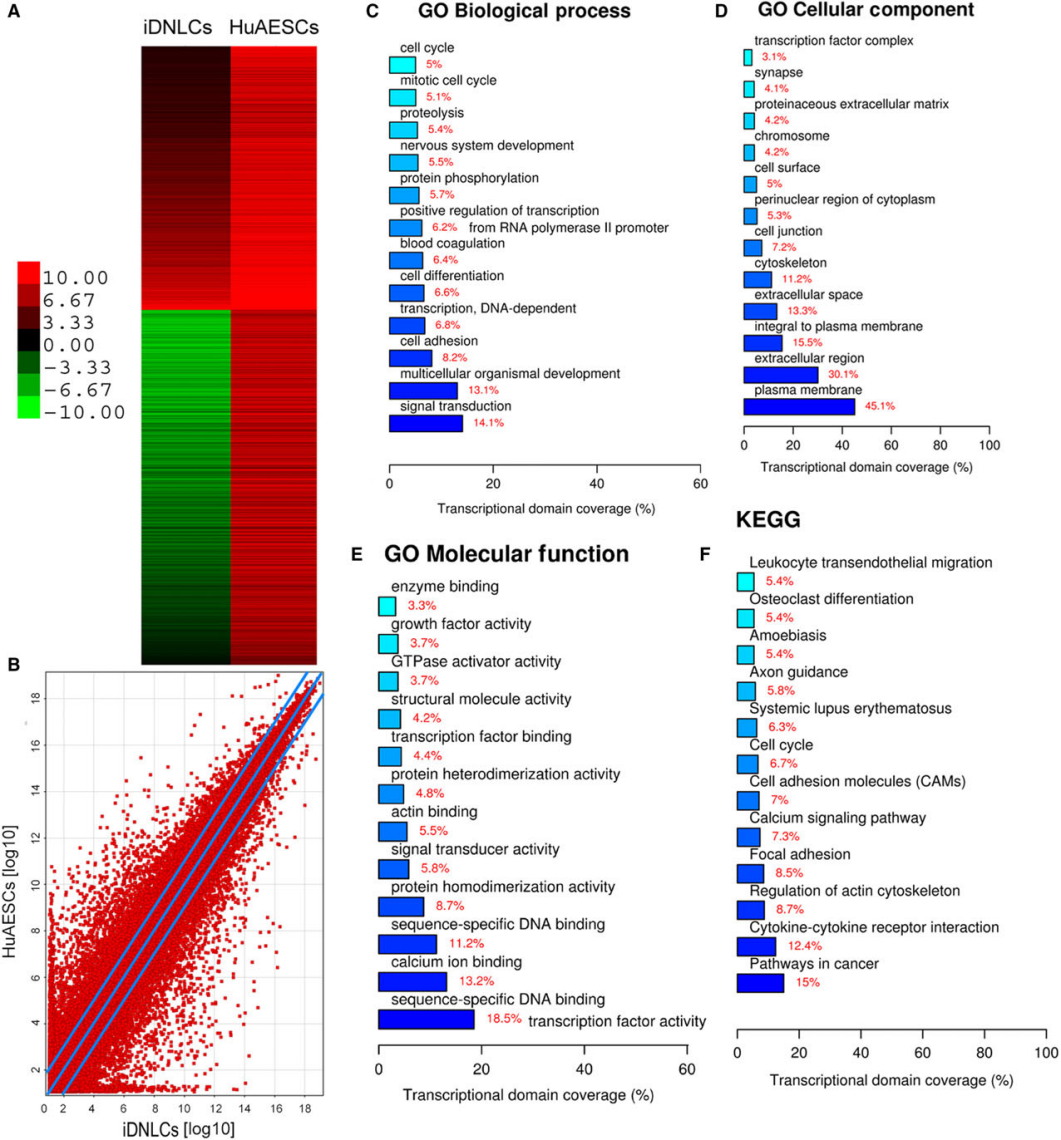
Analysis of differential gene expression profiles before and after the differentiation of HuAESCs (**A**) The heat map shows the results of the microarray analysis of cDNA expression profiles in which the changes in 9871 genes were analysed before and after the induction of HuAESCs. (**B**) The scatter plot shows that the mRNA expression levels of 355 genes were significantly different before and after induction of SPIONs@miR‐141‐HuAESCs into iDNLCs (Log10 [iDNLCs/HuAESCs]>5 indicates a significant up‐regulation; Log10 [iDNLCs/HuAESCs]<‐5 indicates a significant down‐regulation). (**C**) GO analysis found that when iDNLCs and HuAESCs were compared, genes involved in the three biological processes of signal transduction, multicellular organismal development and cell adhesion showed the most significant differential expression. (**D**) In terms of organelle composition, genes whose protein products are typically expressed in the plasma membrane and extracellular regions showed the most significant differential expression. (**E**) In terms of the molecular function of proteins, genes involved in transcription factor activity and sequence‐specific DNA binding showed the most significant differential expression between the two groups. (**F**) According to the Kyoto Encyclopedia of Genes, genes covered by the pathways in cancer and the cytokine–cytokine receptor interaction showed the most significant differential expression between the two groups.

We used cDNA expression microchips and analysed the changes of a total of 9871 gene expression profiles (Fig. 6A and B, Table S2). In all, 355 genes showed significant changes in terms of their mRNA expression levels before and after induced differentiation of SPIONs@miR‐141‐HuAESCs into iDNLCs (Log10 [iDNLCs/HuAESCs]>5 indicated significant up‐regulation; Log10 [iDNLCs/HuAESCs]<‐5 indicated significant down‐regulation). GO analysis showed that when iDNLCs were compared with HuAESCs, genes involved in the three biological processes of signal transduction, multicellular organismal development and cell adhesion showed the most significant differential expression (Fig. 6C). In terms of organelle composition, genes whose protein products are typically located in the plasma membrane and extracellular regions showed the most significant differential expression (Fig. 6D). In terms of the molecular function of proteins, genes involved in transcription factor activity and sequence‐specific DNA binding showed the most significant differential expression between the two groups (Fig. 6E). According to the Kyoto Encyclopedia of Genes, genes covered by the pathways in cancer and cytokine–cytokine receptor interaction showed the most significant differential expression between the two groups (Fig. 6F).

**Figure 7:**
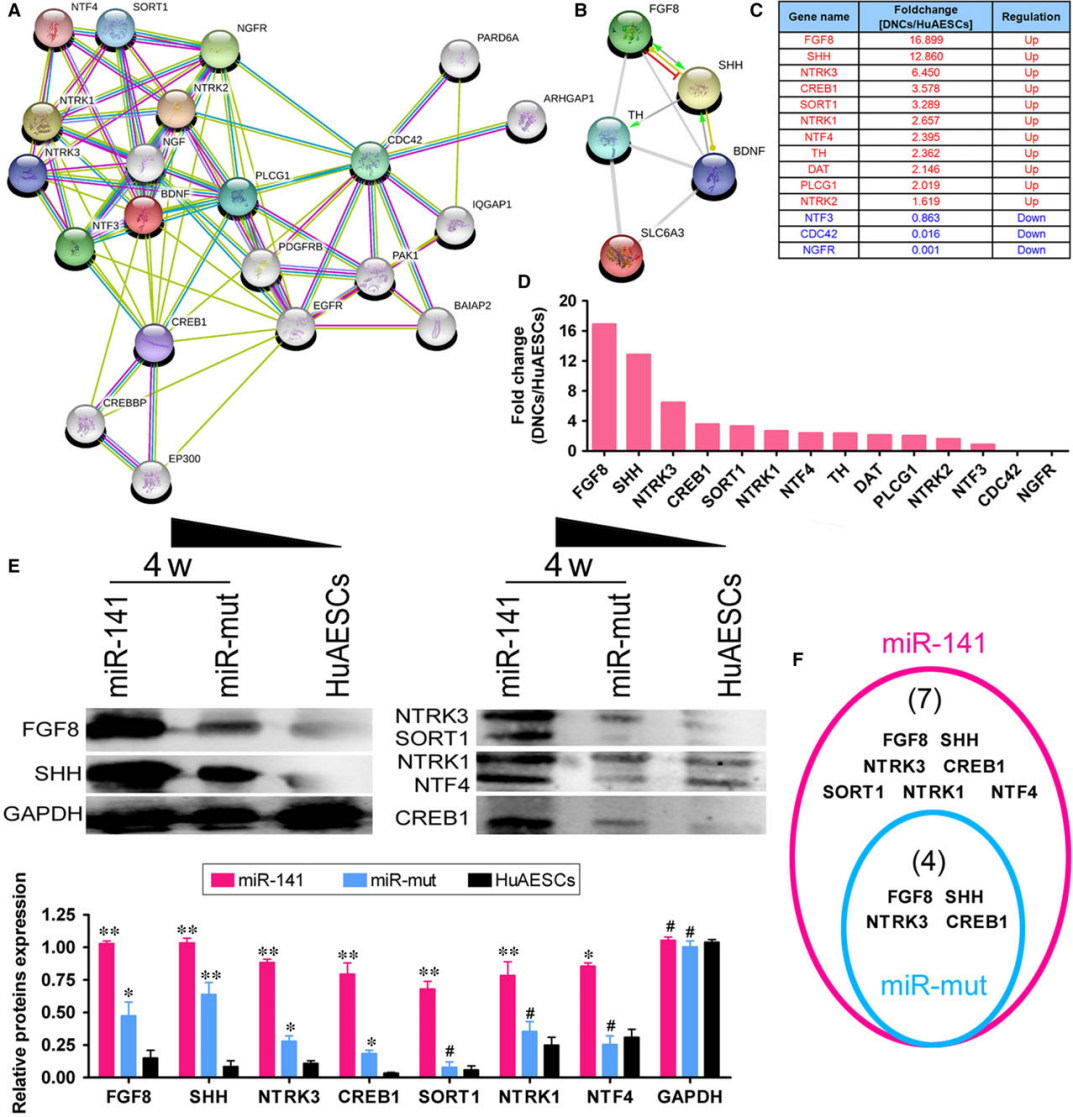
miR‐141 promoted the expression of BDNF and dopaminergic neuron‐associated proteins (**A**) A protein network that interacts with the BDNF protein was identified using STRING: functional protein association networks. (**B**) The STRING tool revealed that BDNF directly promoted the expression of SHH and indirectly promoted the expression of TH and FBF8. (**C**) and (**D**) Comparison with the results of the gene chip assay revealed that except for NTF3, NGFR and CDC42, the mRNA levels of proteins that interact directly or indirectly with the BDNF protein were all significantly increased in iDNLCs cells. (**E**) Western blot analysis showed that the expression levels of FGF, SHH, NTRK3, CREB1, SORT1, NTRK1 and NTF4 in iDNLCs derived from SPIONs@miR‐141‐HuAESCs were significantly higher than the corresponding levels in HuAESCs; **P < 0.01 *vs*. HuAESCs, *n* = 3; *P < 0.05 *vs*. HuAESCs, *n* = 3; ^#^ *P* > 0.05 *vs*. HuAESCs, *n* = 3. (F) In iDNLCs derived from SPIONs@miR‐Mut‐HuAESCs, the expression levels of four factors (FGF, SHH, NTRK3 and CREB1) were significantly higher than those in HuAESCs.

We identified the protein network that interacts with BDNF protein *via* a bioinformatic analysis (STRING: functional protein association networks; https://string-db.org/) (Fig. 7A). The structure predicted by the software suggested that several proteins that interact with the BDNF protein are associated with nerve growth, such as NGF, NTF3/4 and NGFR. Furthermore, we used the above software to analyse the relationship between several factors associated with dopaminergic neurons and BDNF. The prediction software revealed that BDNF directly promoted the expression of SHH and indirectly promoted the expression of TH and FBF8 (Fig. 7B). Compared with the results of gene chips, with the exception of NTF3, NGFR and CDC42, the mRNA levels of proteins that interact directly or indirectly with the BDNF protein were all significantly increased in iDNLCs cells (Fig. 7C and D). Finally, we compared the expression of dopaminergic neuron differentiation‐related factors in iDNLCs derived from the two different sources. Western blot analysis showed that, compared with the levels in HuAESCs, the expression levels of seven factors, FGF, SHH, NTRK3, CREB1, SORT1, NTRK1 and NTF4, were significantly higher in iDNLCs derived from SPIONs@miR‐141‐HuAESCs (Fig. 7E and F). However, in iDNLCs derived from SPIONs@miR‐Mut‐HuAESCs, the expression levels of four factors (FGF, SHH, NTRK3 and CREB1) were significantly higher than the corresponding levels in HuAESCs (Fig. 7E and F). Therefore, the experimental results demonstrate that overexpression of miR‐141 can effectively promote the expression of dopaminergic neuron differentiation‐related factors.

## Discussion

As amniotic epithelial stem cells exhibit characteristics such as pluripotency, immunosuppressive function and trophoblast effects, increasingly more research groups have attempted their secondary development and utilization 1, 2, 3, 4, 15. Our team has conducted a number of studies on HuAESCs, and we have confirmed both *in vivo* and *in vitro* that the stem cell characteristics of these cells can provide new approaches to cell therapy 2, 4. Therefore, ways in which we can more effectively induce their differentiation into specific adult cells have become the focus of our current research. Dopaminergic neurons are significant and essential for the regulation of motor function 13, 14. Investigation of the efficient differentiation of HuAESCs into iDNLCs will provide a wide range of seed cell sources for the treatment of neurodegenerative diseases such as Parkinson's disease. Considering that neuronal growth is inseparable from the stimulation and maintenance provided by neuronal growth factors, we focused on the expression of neuronal growth factors in our study of the differentiation of HuAESCs into iDNLCs.

Histone methylation is a type of epigenetic modification and refers to methylation of N‐terminal arginine or lysine residues in H3 and H4 histones. Its function is primarily manifested in the heterogeneity of chromatin formation, gene imprinting, X chromosome inactivation and transcriptional regulation 5, 16, 17. At present, 24 histone methylation sites have been found, and these are mainly located on lysine residues. Methylation can be manifested as either monomethylation, demethylation or trimethylation and may be mediated by histone methyltransferases or histone demethylases. Methylation of different lysine sites has different effects on gene transcription. Methylation of the H3K4, H3K36 and H3K79 loci can activate gene transcription, whereas methylation of the H3K9, H3K27, H3K79 and H4K20 loci can inhibit transcription 5, 16, 17. We have previously found that in kidney cancer cells, HOTAIR can affect the expression of the cell cycle‐associated proteins p53, p21 and p16 at the transcriptional level *via* the binding of HOTAIR to EZH2 and H3K27me3 8. In addition, in a study of glioma stem cells, we also found that HOTAIR can recruit and enrich EZH2 and LSD1 proteins. EZH2 can keep histone H3K27 in the PDCD4 promoter region in a trimethylated state, and LSD1, as a histone deacetylase, can mediate the demethylation of H3K4me2, while the combined action of both leads to the transcriptional inhibition of PDCD4. siRNA‐mediated silencing of endogenous HOTAIR expression can reduce its recruitment of EZH2 and LSD1 proteins, which subsequently activates PDCD4 at the transcriptional level 7. However, in our previous studies, we used specific siRNA to silence the expression of HOTAIR. As the siRNA we used was specifically targeted to HOTAIR, this mimics the natural silencing of its expression. However, studies on endogenous microRNAs that target the regulation of HOTAIR expression are rare. In this study, we conducted an in‐depth study of miR‐141 in an effort to confirm that it could silence endogenous HOTAIR expression just as microRNAs target and silence mRNA expression.

In addition, in previous studies, liposomes have been commonly used as the mediator for the transfection of cells with plasmid DNA, which takes into account the lipid bilayer structure of eukaryotic cell membranes. Although this strategy has been used for many years, liposomes still have a certain toxicity, and their transfection efficiency is not ideal 7, 10, 11, 12. With the progression of nanomaterials research, some studies have reported the use of nanoparticles as carriers for drugs or for cellular transfection of nucleic acids 7, 10, 11, 12. In this study, we used SPIONs as the medium for cellular transfection of miR‐141. The transfected cells were then analysed by transmission electron microscopy, which revealed high‐density signals of SPIONs in the cells. Northern blot analysis showed that when HuAESCs were transfected with miR‐141 and induced by SPIONs@miR‐141, the number of differentiated iDNLCs after induction was significantly higher than that in the control group. We also confirmed by a variety of methods that SPIONs efficiently transduced large numbers of miR‐141 molecules into HuAESCs, which led to effective, targeted inhibition of endogenous HOTAIR expression. In view of previous reports, HOTAIR functions as a scaffold in the recruitment of PRC2 (containing EZH2, SUZ12 and EED) and the histone demethylase complex LSD1/REST (CoREST), which catalyses H3K27 methylation and H3K4 demethylation 5, 6, 7, 8, 9. So, the lncRNA‐HOTAIR and PRC2 then function together to inhibit the transcriptional activity of target genes 5, 6, 7, 8, 9. However, we found that mature miR‐141 can complement and fully pair with six bases at a specific site within HOTAIR (1295 bp–1300 bp), which suggests that HOTAIR may be a target of miR‐141. And further research showed that overexpression of miR‐141 inhibits the expression of endogenous HOTAIR in HuAESCs during induction. When HOTAIR was cleavaged by miR‐141, the degradation of HOTAIR was triggered. The integrity of the nucleic acid sequence of the ordered lncRNA‐HOTAIR was destroyed, so it cannot recruit the PRC2 complex again. And, we could find that the frequency at which lncRNA‐HOTAIR bound to EZH2 in the nucleus was significantly decreased as the induction time increased in iDNLCs derived from SPIONs@miR‐141‐HuAESCs. Meanwhile, ChIP results showed that in iDNLCs derived from SPIONs@miR‐141‐HuAESCs, the level of EZH2 bound to the BDNF gene promoter region was significantly decreased as the induction time increased. When the expression of HOTAIR was decreased, the level of histone H3K27me3 decreased, while the level of H3K4me2 increased, which led to the direct transcriptional activation of the BDNF gene. Furthermore, we found that when HuAESCs overexpressed miR‐141, not only the expression of BDNF itself but also the expression levels of proteins that directly or indirectly interact with BDNF were elevated. These proteins could stimulate the induced differentiation of HuAESCs into iDNLCs. Through our detailed study, we found that while HOTAIR is a lncRNA, it can still be regulated by microRNAs; this means that mRNA is not the only target of microRNAs. The expression of the target lncRNA‐HOTAIR can still be significantly decreased upon the high intracellular expression of miR‐141 mediated by the nanomaterial SPIONs. Thus, the above phenomena suggest that we can alter the efficiency of stem cell differentiation by manipulating the expression of a particular microRNA or several microRNAs that silence the expression of specific target lncRNAs.

In conclusion, we found an RNA‐DNA regulatory axis, the miR‐141‐HOTAIR‐BDNF axis. The overexpression of miR‐141 effectively inhibited the expression of endogenous lncRNA‐HOTAIR, promoted the transcription and expression of the BDNF gene and ultimately induced amniotic epithelial stem cells to differentiate into iDNLCs. All this resulted from the SPION‐mediated entry of miR‐141 into cells. Thus, this specific nanomaterials an excellent, important, and potential medium that can be used for gene therapy.

## Conflict of Interest

We declared no potential conflicts of interest.

## Supporting information


**Table S1** Antibodies

**Table S2** microArray analysis of mRNAs expression in the iDNLCs and HuAESCs
