# Supplementary material for: SPION‐mediated miR‐141 promotes the differentiation of HuAESCs into dopaminergic neuron‐like cells via suppressing lncRNA‐HOTAIR
Source: J Cell Mol Med. 2018 Feb 7;22(4):2299–310. doi: 10.1111/jcmm.13512 (PMC5867164; doi:10.1111/jcmm.13512)
Supplement: Supplementary file 1 — Table S1 Antibodies [file JCMM-22-2299-s001.docx]

**Table S1 Antibodies**

| Catalog no. | Name | Application and Dilution | Company |
| --- | --- | --- | --- |
| ab129991 | Mouse anti-Human Tyrosine Hydroxylase Ab | WB (1:1000)  IF (1:200) | Abcam (Cambridge, MA, USA) |
| ab133693 | Rabbit anti-human Dopamine Transporter Ab | WB (1:1000)  IF (1:200) | Abcam (Cambridge, MA, USA) |
| ab108319 | Rabbit anti-human BDNF Ab | WB (1:1000) | Abcam (Cambridge, MA, USA) |
| ab18207 | Rabbit anti-human beta III Tubulin Ab | IF (1:200) | Abcam (Cambridge, MA, USA) |
| ab181603 | Rabbit anti-human GAPDH Ab | WB (1:1000) | Abcam (Cambridge, MA, USA) |
| ab191250 | Rabbit anti-human EZH2 Ab | ChIP (1:100)  RIP (1:100) | Abcam (Cambridge, MA, USA) |
| ab81384 | Rabbit anti-human FGF8 Ab | WB (1:1000) | Abcam (Cambridge, MA, USA) |
| ab53281 | Rabbit anti-human Sonic Hedgehog Ab | WB (1:1000) | Abcam (Cambridge, MA, USA) |
| ab135810 | Rabbit anti-human TrkC Ab | WB (1:1000) | Abcam (Cambridge, MA, USA) |
| ab188586 | Rabbit anti-human Sortilin Ab | WB (1:1000) | Abcam (Cambridge, MA, USA) |
| ab76291 | Rabbit anti-human TrkA Ab | WB (1:1000) | Abcam (Cambridge, MA, USA) |
| ab150437 | Rabbit anti-human Neurotrophin 4 Ab | WB (1:1000) | Abcam (Cambridge, MA, USA) |
| ab32515 | Rabbit anti-human CREB Ab | WB (1:1000) | Abcam (Cambridge, MA, USA) |
| ab6785 | Goat Anti-Mouse IgG H&L (FITC) Ab | IF (1:200) | Abcam (Cambridge, MA, USA) |
| ab6717 | Goat Anti-Rabbit IgG H&L (FITC) Ab | IF (1:200) | Abcam (Cambridge, MA, USA) |
| ab6789 | Goat Anti-Mouse IgG H&L (HRP) | WB (1:1000) | Abcam (Cambridge, MA, USA) |
| ab6728 | Rabbit Anti-Mouse IgG H&L (HRP) | WB (1:1000) | Abcam (Cambridge, MA, USA) |
