# Supplementary material for: SPION‐mediated miR‐141 promotes the differentiation of HuAESCs into dopaminergic neuron‐like cells via suppressing lncRNA‐HOTAIR
Source: J Cell Mol Med. 2018 Feb 7;22(4):2299–310. doi: 10.1111/jcmm.13512 (PMC5867164; doi:10.1111/jcmm.13512)
Supplement: Supplementary file 2 — Table S2 microArray analysis of mRNAs expression in the iDNLCs and HuAESCs [file JCMM-22-2299-s002.doc]

**Table S2 microArray analysis of mRNAs expression in the dopaminergic neuron-like cells (iDNL**Cs) and HuAESCs

| **Genbank Accession** | **Gene Symbol** | **Fold change**  **[Log10 (iDNLCs/HuAESCs)]** | **Gene Name** | **Genomic Coordinates (bp)** |
| --- | --- | --- | --- | --- |
| NM_002353 | TACSTD2 | 12.628391 | tumor-associated calcium signal transducer 2 | chr1:59041468-59041409 |
| NM_001074 | UGT2B7 | 12.362728 | UDP glucuronosyltransferase 2 family, polypeptide B7 | chr4:69978384-69978443 |
| NM_003810 | TNFSF10 | 12.123617 | tumor necrosis factor (ligand) superfamily, member 10 | chr3:172224285-172224226 |
| NM_001008 | RPS4Y1 | 11.690547 | ribosomal protein S4, Y-linked 1 | chrY:2722790-2733165 |
| NM_014696 | GPRIN2 | 10.416379 | G protein regulated inducer of neurite outgrowth 2 | chr10:47000509-47000568 |
| NM_174900 | ZFP42 | 10.339309 | ZFP42 zinc finger protein | chr4:188925963-188926022 |
| NM_002371 | MAL | 10.201458 | mal, T-cell differentiation protein | chr2:95719595-95719654 |
| NM_000758 | CSF2 | 9.991797 | colony stimulating factor 2 (granulocyte-macrophage) | chr5:131411452-131411511 |
| NM_001113207 | TSTD1 | 9.925721 | thiosulfate sulfurtransferase (rhodanese)-like domain containing 1 | chr1:161007823-161007764 |
| NM_021102 | SPINT2 | 9.791671 | serine peptidase inhibitor, Kunitz type, 2 | chr19:38782859-38782918 |
| NM_003264 | TLR2 | 9.744069 | toll-like receptor 2 | chr4:154626406-154626465 |
| NM_005558 | LAD1 | 9.621016 | ladinin 1 | chr1:201350401-201350342 |
| NM_001039567 | RPS4Y2 | 9.608575 | ribosomal protein S4, Y-linked 2 | chrY:22941435-22941494 |
| NM_003988 | PAX2 | 9.462859 | paired box 2 | chr10:102589603-102589662 |
| NM_025130 | HKDC1 | 9.378366 | hexokinase domain containing 1 | chr10:71026958-71027017 |
| NM_000600 | IL6 | 9.050206 | interleukin 6 | chr7:22771207-22771266 |
| NM_004654 | USP9Y | 9.038415 | ubiquitin specific peptidase 9, Y-linked | chrY:14972382-14972441 |
| NM_001717 | BNC1 | 9.011982 | basonuclin 1 | chr15:83925019-83924960 |
| NM_016356 | DCDC2 | 8.840479 | doublecortin domain containing 2 | chr6:24172239-24172180 |
| NM_003810 | TNFSF10 | 8.795794 | tumor necrosis factor (ligand) superfamily, member 10 | chr3:172223925-172223866 |
| NM_024490 | ATP10A | 8.786911 | ATPase, class V, type 10A | chr15:25923934-25923875 |
| NM_006147 | IRF6 | 8.748289 | interferon regulatory factor 6 | chr1:209963022-209961941 |
| NM_153360 | APCDD1L | 8.737961 | adenomatosis polyposis coli down-regulated 1-like | chr20:57034568-57034509 |
| NM_005434 | MALL | 8.647757 | mal, T-cell differentiation protein-like | chr2:110841931-110841872 |
| NM_003221 | TFAP2B | 8.469682 | transcription factor AP-2 beta (activating enhancer binding protein 2 beta) | chr6:50815178-50815237 |
| NM_053277 | CLIC6 | 8.36966 | chloride intracellular channel 6 | chr21:36090152-36090211 |
| NM_033256 | PPP1R14A | 8.369461 | protein phosphatase 1, regulatory (inhibitor) subunit 14A | chr19:38743594-38743535 |
| NM_001122665 | DDX3Y | 8.203122 | DEAD (Asp-Glu-Ala-Asp) box helicase 3, Y-linked | chrY:15032230-15032289 |
| NM_201525 | GPR56 | 8.183888 | G protein-coupled receptor 56 | chr16:57698776-57698835 |
| NM_014978 | SORCS3 | 8.171479 | sortilin-related VPS10 domain containing receptor 3 | chr10:107024736-107024795 |
| NM_024337 | IRX1 | 8.144785 | iroquois homeobox 1 | chr5:3601438-3601497 |
| NM_000853 | GSTT1 | 8.102331 | glutathione S-transferase theta 1 | chr22:24376392-24376333 |
| NM_001073 | UGT2B11 | 8.081091 | UDP glucuronosyltransferase 2 family, polypeptide B11 | chr4:70074162-70074103 |
| NM_024572 | GALNT14 | 8.076178 | polypeptide N-acetylgalactosaminyltransferase 14 | chr2:31133429-31133370 |
| NM_152864 | NKAIN4 | 7.9265656 | Na+/K+ transporting ATPase interacting 4 | chr20:61873965-61873906 |
| NM_002354 | EPCAM | 7.884842 | epithelial cell adhesion molecule | chr2:47613894-47613953 |
| BC035312 | TXLNGY | 7.877925 | taxilin gamma pseudogene, Y-linked | chrY:21755964-21756023 |
| NM_014358 | CLEC4E | 7.8477964 | C-type lectin domain family 4, member E | chr12:8685991-8685932 |
| NM_005764 | PDZK1IP1 | 7.817984 | PDZK1 interacting protein 1 | chr1:47650734-47650675 |
| NM_004867 | ITM2A | 7.7687144 | integral membrane protein 2A | chrX:78616934-78616875 |
| NM_024901 | DENND2D | 7.7651534 | DENN/MADD domain containing 2D | chr1:111730006-111729947 |
| NM_001040058 | SPP1 | 7.7061186 | secreted phosphoprotein 1 | chr4:88903920-88903979 |
| NM_000331 | SAA1 | 7.693937 | serum amyloid A1 | chr11:18290868-18291310 |
| NM_001124758 | SPNS2 | 7.6853924 | spinster homolog 2 (Drosophila) | chr17:4442247-4442306 |
| NM_173076 | ABCA12 | 7.683311 | ATP-binding cassette, sub-family A (ABC1), member 12 | chr2:215796336-215796277 |
| NM_033267 | IRX2 | 7.642621 | iroquois homeobox 2 | chr5:2746395-2746336 |
| NM_021195 | CLDN6 | 7.6313562 | claudin 6 | chr16:3065314-3065255 |
| NM_000888 | ITGB6 | 7.6041837 | integrin, beta 6 | chr2:160964233-160958330 |
| NM_001305 | CLDN4 | 7.595352 | claudin 4 | chr7:73246907-73246966 |
| NM_033267 | IRX2 | 7.4470487 | iroquois homeobox 2 | chr5:2746939-2746880 |
| NM_153840 | GPR110 | 7.3877683 | G protein-coupled receptor 110 | chr6:46967904-46967845 |
| NM_007256 | SLCO2B1 | 7.372812 | solute carrier organic anion transporter family, member 2B1 | chr11:74917103-74917162 |
| NM_006043 | HS3ST2 | 7.370882 | heparan sulfate (glucosamine) 3-O-sulfotransferase 2 | chr16:22927193-22927252 |
| NM_004681 | EIF1AY | 7.34244 | eukaryotic translation initiation factor 1A, Y-linked | chrY:22754620-22754679 |
| NM_001136534 | TMEM233 | 7.2997303 | transmembrane protein 233 | chr12:120078559-120078618 |
| NM_003466 | PAX8 | 7.285055 | paired box 8 | chr2:113973634-113973575 |
| NM_001285486 | NEURL3 | 7.2224145 | neuralized E3 ubiquitin protein ligase 3 | chr2:97163478-97163419 |
| NM_198148 | CPXM2 | 7.197399 | carboxypeptidase X (M14 family), member 2 | chr10:125505253-125505194 |
| NM_170741 | KCNJ16 | 7.173001 | potassium inwardly-rectifying channel, subfamily J, member 16 | chr17:68131523-68131582 |
| NM_000594 | TNF | 7.144659 | tumor necrosis factor | chr6:31545837-31545896 |
| NM_152864 | NKAIN4 | 7.1405263 | Na+/K+ transporting ATPase interacting 4 | chr20:61872198-61872139 |
| NR_045129 | TXLNGY | 7.1403346 | taxilin gamma pseudogene, Y-linked | chrY:21749188-21749247 |
| NM_002341 | LTB | 7.1355314 | lymphotoxin beta (TNF superfamily, member 3) | chr6:31548394-31548335 |
| NM_153046 | TDRD9 | 7.104329 | tudor domain containing 9 | chr14:104518565-104518624 |
| NM_001145850 | PROM1 | 7.0545015 | prominin 1 | chr4:15982105-15982046 |
| NM_152611 | LRRN4 | 7.023057 | leucine rich repeat neuronal 4 | chr20:6022354-6022295 |
| NM_004433 | ELF3 | 6.987954 | E74-like factor 3 (ets domain transcription factor, epithelial-specific ) | chr1:201984429-201984488 |
| NM_007193 | ANXA10 | 6.9838047 | annexin A10 | chr4:169108564-169108623 |
| NM_001511 | CXCL1 | 6.970625 | chemokine (C-X-C motif) ligand 1 (melanoma growth stimulating activity, alpha) | chr4:74736850-74736909 |
| NM_184086 | TRIM55 | 6.8431454 | tripartite motif containing 55 | chr8:67064697-67064756 |
| NM_178181 | CDCP1 | 6.8277073 | CUB domain containing protein 1 | chr3:45153600-45152300 |
| NM_020661 | AICDA | 6.8249073 | activation-induced cytidine deaminase | chr12:8755209-8755150 |
| NM_001162997 | SMIM6 | 6.817289 | small integral membrane protein 6 | chr17:73643577-73643636 |
| NR_045128 | TXLNGY | 6.7657166 | taxilin gamma pseudogene, Y-linked | chrY:21767158-21767217 |
| NM_198465 | NRK | 6.737347 | Nik related kinase | chrX:105202523-105202582 |
| NM_017594 | DIRAS2 | 6.734274 | DIRAS family, GTP-binding RAS-like 2 | chr9:93372980-93372921 |
| NR_003948 | HCG22 | 6.7198544 | HLA complex group 22 | chr6:31027543-31027602 |
| NM_006228 | PNOC | 6.697784 | prepronociceptin | chr8:28200772-28200831 |
| NM_152304 | RAB42 | 6.695776 | RAB42, member RAS oncogene family | chr1:28920713-28920772 |
| NM_173078 | SLITRK4 | 6.6716566 | SLIT and NTRK-like family, member 4 | chrX:142716215-142716156 |
| XM_005265702 | UGT2B7 | 6.6595354 | UDP glucuronosyltransferase 2 family, polypeptide B7 | chr4:69955255-69955314 |
| NM_022842 | CDCP1 | 6.624896 | CUB domain containing protein 1 | chr3:45123898-45123839 |
| NM_170736 | KCNJ15 | 6.5868096 | potassium inwardly-rectifying channel, subfamily J, member 15 | chr21:39673353-39673412 |
| NM_003411 | ZFY | 6.5536947 | zinc finger protein, Y-linked | chrY:2849820-2849879 |
| NM_022475 | HHIP | 6.503405 | hedgehog interacting protein | chr4:145658929-145658988 |
| NM_001078 | VCAM1 | 6.502312 | vascular cell adhesion molecule 1 | chr1:101203783-101203842 |
| NM_007365 | PADI2 | 6.488494 | peptidyl arginine deiminase, type II | chr1:17410269-17409137 |
| NM_000576 | IL1B | 6.445627 | interleukin 1, beta | chr2:113587488-113587429 |
| NM_004118 | FOXS1 | 6.4116783 | forkhead box S1 | chr20:30432163-30432104 |
| NM_033058 | TRIM55 | 6.4097214 | tripartite motif containing 55 | chr8:67087190-67087249 |
| NM_021146 | ANGPTL7 | 6.3963656 | angiopoietin-like 7 | chr1:11255859-11255918 |
| NM_012464 | TLL1 | 6.393607 | tolloid-like 1 | chr4:167024085-167024144 |
| NM_007197 | FZD10 | 6.387868 | frizzled class receptor 10 | chr12:130650174-130650233 |
| NM_001073 | UGT2B11 | 6.3799043 | UDP glucuronosyltransferase 2 family, polypeptide B11 | chr4:70066280-70066221 |
| NM_182920 | ADAMTS9 | 6.3598156 | ADAM metallopeptidase with thrombospondin type 1 motif, 9 | chr3:64501648-64501589 |
| NM_053036 | NPFFR2 | 6.318121 | neuropeptide FF receptor 2 | chr4:73013257-73013316 |
| NM_004165 | RRAD | 6.297782 | Ras-related associated with diabetes | chr16:66956234-66956175 |
| NM_058238 | WNT7B | 6.291052 | wingless-type MMTV integration site family, member 7B | chr22:46319002-46318943 |
| NM_004823 | KCNK6 | 6.233229 | potassium channel, subfamily K, member 6 | chr19:38818817-38818876 |
| NM_032528 | ST6GAL2 | 6.198974 | ST6 beta-galactosamide alpha-2,6-sialyltranferase 2 | chr2:107418204-107418145 |
| NM_000909 | NPY1R | 6.1753993 | neuropeptide Y receptor Y1 | chr4:164245435-164245376 |
| NM_030641 | APOL6 | 6.1351266 | apolipoprotein L, 6 | chr22:36057118-36057177 |
| NM_004473 | FOXE1 | 6.1327353 | forkhead box E1 (thyroid transcription factor 2) | chr9:100618916-100618975 |
| NM_012206 | HAVCR1 | 6.1077666 | hepatitis A virus cellular receptor 1 | chr5:156456630-156456571 |
| NM_020826 | SYT13 | 6.0983763 | synaptotagmin XIII | chr11:45262089-45262030 |
| NM_000204 | CFI | 6.0900445 | complement factor I | chr4:110662123-110662064 |
| NM_001956 | EDN2 | 6.086052 | endothelin 2 | chr1:41944515-41944456 |
| NM_002773 | PRSS8 | 6.0753126 | protease, serine, 8 | chr16:31142813-31142754 |
| NM_023915 | GPR87 | 6.050344 | G protein-coupled receptor 87 | chr3:151012341-151012282 |
| NM_152899 | IL4I1 | 6.0306473 | interleukin 4 induced 1 | chr19:50392976-50392917 |
| NM_001282971 | MTSS1 | 6.001178 | metastasis suppressor 1 | chr8:125565128-125565069 |
| NM_019076 | UGT1A8 | 5.9825315 | UDP glucuronosyltransferase 1 family, polypeptide A8 | chr2:234681212-234681271 |
| NM_178497 | C4orf26 | 5.9699144 | chromosome 4 open reading frame 26 | chr4:76489487-76489546 |
| NM_004288 | CYTIP | 5.9692307 | cytohesin 1 interacting protein | chr2:158272226-158272167 |
| NM_021603 | FXYD2 | 5.952287 | FXYD domain containing ion transport regulator 2 | chr11:117691003-117690944 |
| XM_005249745 | IL6 | 5.932899 | interleukin 6 | chr7:22769363-22769422 |
| NM_005459 | GUCA1C | 5.9069214 | guanylate cyclase activator 1C | chr3:108639333-108635053 |
| NM_000433 | NCF2 | 5.8822308 | neutrophil cytosolic factor 2 | chr1:183524784-183524725 |
| NM_001075 | UGT2B10 | 5.870988 | UDP glucuronosyltransferase 2 family, polypeptide B10 | chr4:69696473-69696532 |
| NM_030754 | SAA2 | 5.869875 | serum amyloid A2 | chr11:18266850-18266791 |
| NM_015204 | THSD7A | 5.869141 | thrombospondin, type I, domain containing 7A | chr7:11414524-11414465 |
| NM_000912 | OPRK1 | 5.8680787 | opioid receptor, kappa 1 | chr8:54138687-54138628 |
| NM_004751 | GCNT3 | 5.8433657 | glucosaminyl (N-acetyl) transferase 3, mucin type | chr15:59911364-59911423 |
| NM_001011880 | CLEC18B | 5.8413353 | C-type lectin domain family 18, member B | chr16:74442597-74442538 |
| NM_006033 | LIPG | 5.810498 | lipase, endothelial | chr18:47118360-47118419 |
| NM_153345 | TMEM139 | 5.8021493 | transmembrane protein 139 | chr7:142984122-142984181 |
| NM_145168 | SDR42E1 | 5.799032 | short chain dehydrogenase/reductase family 42E, member 1 | chr16:82031603-82031544 |
| NM_001002919 | FAM150B | 5.7767787 | family with sequence similarity 150, member B | chr2:279994-279935 |
| NM_139161 | CRB3 | 5.7632675 | crumbs family member 3 | chr19:6467158-6467217 |
| NM_002298 | LCP1 | 5.6884356 | lymphocyte cytosolic protein 1 (L-plastin) | chr13:46700396-46700337 |
| NM_018891 | LAMC2 | 5.630951 | laminin, gamma 2 | chr1:183209301-183209453 |
| NM_001252 | CD70 | 5.611721 | CD70 molecule | chr19:6585926-6585867 |
| NM_032023 | RASSF4 | 5.6001425 | Ras association (RalGDS/AF-6) domain family member 4 | chr10:45489768-45489827 |
| NM_014464 | TINAG | 5.598611 | tubulointerstitial nephritis antigen | chr6:54185380-54185439 |
| NM_014893 | NLGN4Y | 5.5838118 | neuroligin 4, Y-linked | chrY:16954955-16955014 |
| NM_032413 | C15orf48 | 5.5785007 | chromosome 15 open reading frame 48 | chr15:45725295-45725354 |
| NM_021995 | UTS2 | 5.5729513 | urotensin 2 | chr1:7909705-7907889 |
| NM_004360 | CDH1 | 5.547446 | cadherin 1, type 1, E-cadherin (epithelial) | chr16:68869214-68869273 |
| NM_000475 | NR0B1 | 5.537668 | nuclear receptor subfamily 0, group B, member 1 | chrX:30322698-30322639 |
| NM_001250 | CD40 | 5.5168743 | CD40 molecule, TNF receptor superfamily member 5 | chr20:44755280-44755339 |
| NM_001511 | CXCL1 | 5.4972477 | chemokine (C-X-C motif) ligand 1 (melanoma growth stimulating activity, alpha) | chr4:74735646-74735705 |
| NM_004654 | USP9Y | 5.4967046 | ubiquitin specific peptidase 9, Y-linked | chrY:14971489-14971548 |
| NM_178470 | DCAF12L1 | 5.4916053 | DDB1 and CUL4 associated factor 12-like 1 | chrX:125683482-125683423 |
| NM_001935 | DPP4 | 5.4869885 | dipeptidyl-peptidase 4 | chr2:162849045-162848986 |
| NM_198239 | WISP3 | 5.4740515 | WNT1 inducible signaling pathway protein 3 | chr6:112389473-112389532 |
| NM_002487 | NDN | 5.4635625 | necdin, melanoma antigen (MAGE) family member | chr15:23931114-23931055 |
| NM_004049 | BCL2A1 | 5.4624023 | BCL2-related protein A1 | chr15:80263195-80263136 |
| NM_000273 | GPR143 | 5.4540024 | G protein-coupled receptor 143 | chrX:9693758-9693699 |
| NM_000737 | CGB | 5.445443 | chorionic gonadotropin, beta polypeptide | chr19:49526300-49526241 |
| NM_003385 | VSNL1 | 5.4435396 | visinin-like 1 | chr2:17837224-17837283 |
| NM_020768 | KCTD16 | 5.4363427 | potassium channel tetramerization domain containing 16 | chr5:143853617-143853676 |
| NM_153212 | GJB4 | 5.4329805 | gap junction protein, beta 4, 30.3kDa | chr1:35227853-35227912 |
| NM_152365 | KDF1 | 5.425951 | keratinocyte differentiation factor 1 | chr1:27276181-27276122 |
| NM_152869 | RGN | 5.421091 | regucalcin | chrX:46952536-46952595 |
| NM_001034837 | KCNIP1 | 5.413887 | Kv channel interacting protein 1 | chr5:170163016-170163075 |
| NM_144777 | SCEL | 5.390442 | sciellin | chr13:78218564-78218623 |
| NM_025216 | WNT10A | 5.3690796 | wingless-type MMTV integration site family, member 10A | chr2:219758393-219758452 |
| AK124396 | HHIP | 5.3584514 | hedgehog interacting protein | chr4:145666336-145666395 |
| NM_015714 | G0S2 | 5.354432 | G0/G1 switch 2 | chr1:209849597-209849656 |
| NM_017852 | NLRP2 | 5.3544116 | NLR family, pyrin domain containing 2 | chr19:55505674-55505733 |
| NM_194284 | CLDN23 | 5.3464575 | claudin 23 | chr8:8561413-8561472 |
| NM_006871 | RIPK3 | 5.343291 | receptor-interacting serine-threonine kinase 3 | chr14:24806583-24806524 |
| NM_000211 | ITGB2 | 5.331863 | integrin, beta 2 (complement component 3 receptor 3 and 4 subunit) | chr21:46305985-46305926 |
| NM_005562 | LAMC2 | 5.324197 | laminin, gamma 2 | chr1:183213610-183213669 |
| NM_014220 | TM4SF1 | 5.3122153 | transmembrane 4 L six family member 1 | chr3:149093493-149093343 |
| NM_032528 | ST6GAL2 | 5.292208 | ST6 beta-galactosamide alpha-2,6-sialyltranferase 2 | chr2:107450557-107449116 |
| NM_138786 | TM4SF18 | 5.2694044 | transmembrane 4 L six family member 18 | chr3:149040112-149040053 |
| NM_001080521 | RASSF10 | 5.2650175 | Ras association (RalGDS/AF-6) domain family (N-terminal) member 10 | chr11:13032560-13032619 |
| NR_045128 | TXLNGY | 5.2466264 | taxilin gamma pseudogene, Y-linked | chrY:21766231-21766290 |
| NM_021076 | NEFH | 5.2459354 | neurofilament, heavy polypeptide | chr22:29887194-29887253 |
| NM_004946 | DOCK2 | 5.22273 | dedicator of cytokinesis 2 | chr5:169506070-169506129 |
| BC108702 | NRK | 5.2099633 | Nik related kinase | chrX:105139249-105139308 |
| NM_006169 | NNMT | 5.205883 | nicotinamide N-methyltransferase | chr11:114183072-114183131 |
| NM_000201 | ICAM1 | 5.1997166 | intercellular adhesion molecule 1 | chr19:10396298-10396358 |
| NM_006690 | MMP24 | 5.1888485 | matrix metallopeptidase 24 (membrane-inserted) | chr20:33864708-33864767 |
| NM_001785 | CDA | 5.1295195 | cytidine deaminase | chr1:20945069-20945128 |
| NM_020873 | LRRN1 | 5.121582 | leucine rich repeat neuronal 1 | chr3:3888753-3888812 |
| NM_001164238 | NLGN4Y | 5.104051 | neuroligin 4, Y-linked | chrY:16845337-16845396 |
| NM_005020 | PDE1C | 5.0999317 | phosphodiesterase 1C, calmodulin-dependent 70kDa | chr7:31855645-31855586 |
| NM_001010848 | NRG3 | 5.095309 | neuregulin 3 | chr10:84746874-84746933 |
| NM_014583 | LMCD1 | 5.091036 | LIM and cysteine-rich domains 1 | chr3:8609688-8609747 |
| NM_001629 | ALOX5AP | 5.0794473 | arachidonate 5-lipoxygenase-activating protein | chr13:31338155-31338214 |
| NM_005472 | KCNE3 | 5.0620627 | potassium voltage-gated channel, Isk-related family, member 3 | chr11:74166545-74166486 |
| NM_001080471 | PEAR1 | 5.052977 | platelet endothelial aggregation receptor 1 | chr1:156885677-156885736 |
| NM_005181 | CA3 | 5.0408483 | carbonic anhydrase III, muscle specific | chr8:86360865-86360924 |
| NM_001257 | CDH13 | 5.0405264 | cadherin 13 | chr16:83830027-83830086 |
| NM_003189 | TAL1 | 5.0195723 | T-cell acute lymphocytic leukemia 1 | chr1:47682477-47682418 |
| NM_002299 | LCT | 5.0176244 | lactase | chr2:136545946-136545887 |
| NM_014751 | MTSS1 | 5.0094604 | metastasis suppressor 1 | chr8:125563314-125563255 |
| NM_014237 | ADAM18 | 5.00801 | ADAM metallopeptidase domain 18 | chr8:39581412-39587461 |
| NM_001286233 | SLC2A14 | -5.045224 | solute carrier family 2 (facilitated glucose transporter), member 14 | chr12:7965262-7965203 |
| NM_000867 | HTR2B | -5.0697184 | 5-hydroxytryptamine (serotonin) receptor 2B, G protein-coupled | chr2:231973300-231973241 |
| NM_001718 | BMP6 | -5.072348 | bone morphogenetic protein 6 | chr6:7881395-7881454 |
| AB058691 | ALX4 | -5.092354 | ALX homeobox 4 | chr11:44282055-44281996 |
| NM_001098722 | GNG4 | -5.095523 | guanine nucleotide binding protein (G protein), gamma 4 | chr1:235714443-235714384 |
| NM_014368 | LHX6 | -5.0996094 | LIM homeobox 6 | chr9:124965139-124965080 |
| NM_001008223 | C1QL4 | -5.1230745 | complement component 1, q subcomponent-like 4 | chr12:49726319-49726260 |
| NM_001584 | MPPED2 | -5.1277046 | metallophosphoesterase domain containing 2 | chr11:30431949-30431890 |
| NM_022970 | FGFR2 | -5.138611 | fibroblast growth factor receptor 2 | chr10:123243276-123243217 |
| NM_002606 | PDE9A | -5.139785 | phosphodiesterase 9A | chr21:44188338-44188397 |
| NM_006684 | CFHR4 | -5.1638317 | complement factor H-related 4 | chr1:196884181-196884240 |
| NM_021973 | HAND2 | -5.1698904 | heart and neural crest derivatives expressed 2 | chr4:174448332-174448273 |
| NM_152709 | STOX1 | -5.1766453 | storkhead box 1 | chr10:70652436-70652495 |
| NM_005994 | TBX2 | -5.190965 | T-box 2 | chr17:59486256-59486315 |
| NM_018242 | SLC47A1 | -5.2019815 | solute carrier family 47 (multidrug and toxin extrusion), member 1 | chr17:19481868-19481927 |
| NM_004349 | RUNX1T1 | -5.2170534 | runt-related transcription factor 1; translocated to, 1 (cyclin D-related) | chr8:92972310-92972251 |
| NM_001190455 | CHRNA7 | -5.23467 | cholinergic receptor, nicotinic, alpha 7 (neuronal) | chr15:32462222-32462281 |
| NM_001017372 | SLC27A6 | -5.2486176 | solute carrier family 27 (fatty acid transporter), member 6 | chr5:128364106-128365299 |
| NM_004172 | SLC1A3 | -5.2533674 | solute carrier family 1 (glial high affinity glutamate transporter), member 3 | chr5:36688204-36688263 |
| NM_001099 | ACPP | -5.2692714 | acid phosphatase, prostate | chr3:132077343-132077402 |
| AK094730 | HRK | -5.2705317 | harakiri, BCL2 interacting protein | chr12:117294035-117293976 |
| NM_001199219 | INMT | -5.276021 | indolethylamine N-methyltransferase | chr7:30797158-30797217 |
| NM_002543 | OLR1 | -5.298411 | oxidized low density lipoprotein (lectin-like) receptor 1 | chr12:10311657-10311598 |
| NM_003391 | WNT2 | -5.3014736 | wingless-type MMTV integration site family member 2 | chr7:116917444-116917385 |
| NM_006329 | FBLN5 | -5.3056164 | fibulin 5 | chr14:92336140-92336081 |
| NM_000612 | IGF2 | -5.3168316 | insulin-like growth factor 2 (somatomedin A) | chr11:2154754-2154402 |
| NM_021570 | BARX1 | -5.3222356 | BARX homeobox 1 | chr9:96714346-96714287 |
| NM_004056 | CA8 | -5.338319 | carbonic anhydrase VIII | chr8:61102510-61102451 |
| NM_001040709 | SYPL2 | -5.3596854 | synaptophysin-like 2 | chr1:110024705-110024764 |
| NM_004626 | WNT11 | -5.374003 | wingless-type MMTV integration site family, member 11 | chr11:75897645-75897586 |
| NM_130385 | MRVI1 | -5.386036 | murine retrovirus integration site 1 homolog | chr11:10594698-10594639 |
| NM_153235 | TXLNB | -5.3874087 | taxilin beta | chr6:139561529-139561470 |
| NM_002563 | P2RY1 | -5.3929567 | purinergic receptor P2Y, G-protein coupled, 1 | chr3:152555738-152555797 |
| NM_152520 | ZNF385B | -5.415714 | zinc finger protein 385B | chr2:180307554-180307495 |
| NM_000738 | CHRM1 | -5.418471 | cholinergic receptor, muscarinic 1 | chr11:62676214-62676155 |
| NM_203422 | LRRN4CL | -5.4207897 | LRRN4 C-terminal like | chr11:62453945-62453886 |
| NM_020927 | VAT1L | -5.422849 | vesicle amine transport 1-like | chr16:78013635-78013694 |
| NM_013447 | EMR2 | -5.4294887 | egf-like module containing, mucin-like, hormone receptor-like 2 | chr19:14843917-14843858 |
| NM_003151 | STAT4 | -5.43392 | signal transducer and activator of transcription 4 | chr2:192011440-192011381 |
| NM_020883 | ZSWIM5 | -5.4451203 | zinc finger, SWIM-type containing 5 | chr1:45482333-45482274 |
| NM_005244 | EYA2 | -5.457301 | eyes absent homolog 2 (Drosophila) | chr20:45817096-45817155 |
| NM_005806 | OLIG2 | -5.45847 | oligodendrocyte lineage transcription factor 2 | chr21:34400374-34400433 |
| NM_022114 | PRDM16 | -5.4592857 | PR domain containing 16 | chr1:3355062-3355121 |
| NM_018942 | HMX1 | -5.5137944 | H6 family homeobox 1 | chr4:8869066-8869007 |
| NM_052836 | CDH23 | -5.528216 | cadherin-related 23 | chr10:73376989-73377048 |
| NM_001198 | PRDM1 | -5.5424185 | PR domain containing 1, with ZNF domain | chr6:106557662-106557721 |
| NM_000237 | LPL | -5.5555835 | lipoprotein lipase | chr8:19824187-19824246 |
| NM_004119 | FLT3 | -5.562189 | fms-related tyrosine kinase 3 | chr13:28578051-28577993 |
| NM_001006658 | CR2 | -5.571588 | complement component (3d/Epstein Barr virus) receptor 2 | chr1:207663116-207663175 |
| NM_001134363 | RBM20 | -5.611003 | RNA binding motif protein 20 | chr10:112598859-112598918 |
| NM_020211 | RGMA | -5.6292815 | repulsive guidance molecule family member a | chr15:93586835-93586776 |
| NM_012082 | ZFPM2 | -5.63118 | zinc finger protein, FOG family member 2 | chr8:106816325-106816384 |
| NM_003026 | SH3GL2 | -5.638135 | SH3-domain GRB2-like 2 | chr9:17796791-17796850 |
| NM_002257 | KLK1 | -5.673624 | kallikrein 1 | chr19:51322506-51322447 |
| NM_021205 | RHOU | -5.678664 | ras homolog family member U | chr1:228882099-228882158 |
| NM_152335 | C15orf27 | -5.6866016 | chromosome 15 open reading frame 27 | chr15:76497139-76497198 |
| NM_002509 | NKX2-2 | -5.701825 | NK2 homeobox 2 | chr20:21491858-21491799 |
| NM_033229 | TRIM15 | -5.71723 | tripartite motif containing 15 | chr6:30140403-30140462 |
| NM_001584 | MPPED2 | -5.745675 | metallophosphoesterase domain containing 2 | chr11:30601929-30601870 |
| NM_152679 | SLC10A4 | -5.7462435 | solute carrier family 10, member 4 | chr4:48490973-48491032 |
| NM_080759 | DACH1 | -5.7642345 | dachshund family transcription factor 1 | chr13:72049898-72049839 |
| NM_018057 | SLC6A15 | -5.7656875 | solute carrier family 6 (neutral amino acid transporter), member 15 | chr12:85277583-85277524 |
| NM_001720 | BMP8B | -5.8118796 | bone morphogenetic protein 8b | chr1:40240526-40240075 |
| NM_133329 | KCNG3 | -5.814078 | potassium voltage-gated channel, subfamily G, member 3 | chr2:42669459-42669400 |
| NM_014178 | STXBP6 | -5.836406 | syntaxin binding protein 6 (amisyn) | chr14:25326267-25325283 |
| XM_006710782 | LRRC7 | -5.8512874 | leucine rich repeat containing 7 | chr1:70144101-70144160 |
| NM_001282394 | WNK2 | -5.8517065 | WNK lysine deficient protein kinase 2 | chr9:96080578-96080637 |
| NM_000926 | PGR | -5.873833 | progesterone receptor | chr11:100912768-100912709 |
| NM_019035 | PCDH18 | -5.8770943 | protocadherin 18 | chr4:138440160-138440101 |
| NM_182767 | SLC6A15 | -5.917197 | solute carrier family 6 (neutral amino acid transporter), member 15 | chr12:85266530-85266471 |
| NM_020404 | CD248 | -5.927871 | CD248 molecule, endosialin | chr11:66082335-66082276 |
| NM_018593 | SLC16A10 | -5.9300756 | solute carrier family 16 (aromatic amino acid transporter), member 10 | chr6:111543660-111543716 |
| NM_175733 | SYT9 | -5.9442105 | synaptotagmin IX | chr11:7489539-7489598 |
| NM_021197 | WFDC1 | -5.9483814 | WAP four-disulfide core domain 1 | chr16:84360556-84362981 |
| NM_006159 | NELL2 | -5.94919 | NEL-like 2 (chicken) | chr12:44902496-44902437 |
| NM_012183 | FOXD3 | -5.9511814 | forkhead box D3 | chr1:63790602-63790661 |
| NM_001452 | FOXF2 | -5.954116 | forkhead box F2 | chr6:1395386-1395445 |
| NM_033343 | LHX4 | -5.95514 | LIM homeobox 4 | chr1:180243753-180243812 |
| NM_015567 | SLITRK5 | -5.966155 | SLIT and NTRK-like family, member 5 | chr13:88331586-88331645 |
| NM_014717 | ZNF536 | -5.9808207 | zinc finger protein 536 | chr19:31040337-31040396 |
| NM_138815 | DPPA2 | -5.9826365 | developmental pluripotency associated 2 | chr3:109012699-109012641 |
| AK310215 | ACTG2 | -5.983008 | actin, gamma 2, smooth muscle, enteric | chr2:74130241-74130300 |
| NM_001884 | HAPLN1 | -5.9839287 | hyaluronan and proteoglycan link protein 1 | chr5:82937382-82937323 |
| NM_000142 | FGFR3 | -5.9854856 | fibroblast growth factor receptor 3 | chr4:1810363-1810422 |
| NM_002193 | INHBB | -5.9886703 | inhibin, beta B | chr2:121108780-121108839 |
| NM_012259 | HEY2 | -5.991352 | hes-related family bHLH transcription factor with YRPW motif 2 | chr6:126082004-126082063 |
| NM_001093726 | SEPP1 | -6.014503 | selenoprotein P, plasma, 1 | chr5:42800881-42800822 |
| NM_001040462 | BTNL8 | -6.042389 | butyrophilin-like 8 | chr5:180377772-180377831 |
| NM_000218 | KCNQ1 | -6.0619535 | potassium voltage-gated channel, KQT-like subfamily, member 1 | chr11:2870117-2870176 |
| NM_001015001 | CKMT1A | -6.078274 | creatine kinase, mitochondrial 1A | chr15:43991282-43991341 |
| NM_173849 | GSC | -6.1196 | goosecoid homeobox | chr14:95234799-95234740 |
| NM_001037341 | PDE4B | -6.1287045 | phosphodiesterase 4B, cAMP-specific | chr1:66839789-66839848 |
| NM_001004354 | NRARP | -6.132168 | NOTCH-regulated ankyrin repeat protein | chr9:140194182-140194123 |
| NM_000612 | IGF2 | -6.1476374 | insulin-like growth factor 2 (somatomedin A) | chr11:2150453-2150394 |
| NM_080647 | TBX1 | -6.1700373 | T-box 1 | chr22:19754788-19754847 |
| NM_002052 | GATA4 | -6.202149 | GATA binding protein 4 | chr8:11617080-11617139 |
| NM_033064 | ATCAY | -6.2050147 | ataxia, cerebellar, Cayman type | chr19:3927951-3928010 |
| NM_182767 | SLC6A15 | -6.2198043 | solute carrier family 6 (neutral amino acid transporter), member 15 | chr12:85254418-85254359 |
| NM_018018 | SLC38A4 | -6.2404385 | solute carrier family 38, member 4 | chr12:47159136-47159077 |
| NM_019845 | RPRM | -6.2611017 | reprimo, TP53 dependent G2 arrest mediator candidate | chr2:154334235-154334176 |
| NM_001615 | ACTG2 | -6.2611628 | actin, gamma 2, smooth muscle, enteric | chr2:74146700-74146759 |
| NM_033132 | ZIC5 | -6.274606 | Zic family member 5 | chr13:100615740-100615681 |
| NM_001164442 | FAM159B | -6.3168516 | family with sequence similarity 159, member B | chr5:64013858-64013917 |
| NM_003178 | SYN2 | -6.31823 | synapsin II | chr3:12227160-12227219 |
| NM_006206 | PDGFRA | -6.355584 | platelet-derived growth factor receptor, alpha polypeptide | chr4:55163989-55164048 |
| NM_006108 | SPON1 | -6.3613663 | spondin 1, extracellular matrix protein | chr11:14289180-14289239 |
| NM_144505 | KLK8 | -6.371526 | kallikrein-related peptidase 8 | chr19:51499375-51499316 |
| NM_002653 | PITX1 | -6.3837996 | paired-like homeodomain 1 | chr5:134363483-134363424 |
| AF498274 | LRRC7 | -6.390785 | leucine rich repeat containing 7 | chr1:70340576-70340635 |
| NM_001719 | BMP7 | -6.484661 | bone morphogenetic protein 7 | chr20:55803439-55803380 |
| NM_003317 | NKX2-1 | -6.49899 | NK2 homeobox 1 | chr14:36985777-36985718 |
| NM_001005463 | EBF3 | -6.5006304 | early B-cell factor 3 | chr10:131633847-131633788 |
| NM_015430 | PAMR1 | -6.5025787 | peptidase domain containing associated with muscle regeneration 1 | chr11:35453900-35453841 |
| NM_016269 | LEF1 | -6.5089154 | lymphoid enhancer-binding factor 1 | chr4:108968805-108968746 |
| NM_021116 | ADCY1 | -6.588032 | adenylate cyclase 1 (brain) | chr7:45762328-45762385 |
| NM_033120 | NKD2 | -6.610753 | naked cuticle homolog 2 (Drosophila) | chr5:1038719-1038778 |
| NM_020794 | LRRC7 | -6.654657 | leucine rich repeat containing 7 | chr1:70589063-70589122 |
| NM_005099 | ADAMTS4 | -6.6621423 | ADAM metallopeptidase with thrombospondin type 1 motif, 4 | chr1:161159705-161159646 |
| NM_005410 | SEPP1 | -6.673027 | selenoprotein P, plasma, 1 | chr5:42799939-42799904 |
| NM_016358 | IRX4 | -6.6781435 | iroquois homeobox 4 | chr5:1877555-1877533 |
| NM_001864 | COX7A1 | -6.679157 | cytochrome c oxidase subunit VIIa polypeptide 1 (muscle) | chr19:36642437-36642378 |
| NM_001265 | CDX2 | -6.679635 | caudal type homeobox 2 | chr13:28536361-28536302 |
| NM_000724 | CACNB2 | -6.682105 | calcium channel, voltage-dependent, beta 2 subunit | chr10:18829909-18829968 |
| NM_004624 | VIPR1 | -6.724802 | vasoactive intestinal peptide receptor 1 | chr3:42578746-42578805 |
| NM_001017534 | CARD16 | -6.7439046 | caspase recruitment domain family, member 16 | chr11:104915286-104915227 |
| NM_002426 | MMP12 | -6.784013 | matrix metallopeptidase 12 (macrophage elastase) | chr11:102733653-102733594 |
| NM_018059 | RADIL | -6.8620358 | Ras association and DIL domains | chr7:4838849-4838790 |
| NM_001007232 | CARD17 | -6.863986 | caspase recruitment domain family, member 17 | chr11:104971256-104970106 |
| NM_003182 | TAC1 | -6.865313 | tachykinin, precursor 1 | chr7:97369454-97369513 |
| NM_001719 | BMP7 | -6.917631 | bone morphogenetic protein 7 | chr20:55745614-55745555 |
| NM_000275 | OCA2 | -6.9303126 | oculocutaneous albinism II | chr15:28000126-28000067 |
| NM_001017424 | KCNK2 | -6.9464173 | potassium channel, subfamily K, member 2 | chr1:215410352-215410411 |
| NM_002425 | MMP10 | -6.9512916 | matrix metallopeptidase 10 (stromelysin 2) | chr11:102641464-102641405 |
| NM_032808 | LINGO1 | -6.9651923 | leucine rich repeat and Ig domain containing 1 | chr15:77906085-77906026 |
| NM_000522 | HOXA13 | -7.0170827 | homeobox A13 | chr7:27236733-27236674 |
| NM_018176 | LGI2 | -7.025251 | leucine-rich repeat LGI family, member 2 | chr4:25000598-25000539 |
| NM_001189 | NKX3-2 | -7.1520944 | NK3 homeobox 2 | chr4:13542835-13542776 |
| NM_031455 | CCDC3 | -7.1554174 | coiled-coil domain containing 3 | chr10:12938688-12938629 |
| NM_017410 | HOXC13 | -7.194611 | homeobox C13 | chr12:54340034-54340093 |
| NM_080759 | DACH1 | -7.2203064 | dachshund family transcription factor 1 | chr13:72012434-72012375 |
| NM_144669 | GLT1D1 | -7.330146 | glycosyltransferase 1 domain containing 1 | chr12:129469286-129469345 |
| NM_001035 | RYR2 | -7.386393 | ryanodine receptor 2 (cardiac) | chr1:237996423-237996482 |
| NM_032229 | SLITRK6 | -7.4602404 | SLIT and NTRK-like family, member 6 | chr13:86368069-86368010 |
| NM_031957 | KRTAP1-5 | -7.5390835 | keratin associated protein 1-5 | chr17:39182367-39182308 |
| NM_020134 | DPYSL5 | -7.5645056 | dihydropyrimidinase-like 5 | chr2:27170005-27170064 |
| NM_001145161 | UBE2QL1 | -7.606993 | ubiquitin-conjugating enzyme E2Q family-like 1 | chr5:6491652-6491711 |
| NM_017551 | GRID1 | -7.6421585 | glutamate receptor, ionotropic, delta 1 | chr10:87361903-87361844 |
| NM_001004317 | LIN28B | -7.6585493 | lin-28 homolog B (C. elegans) | chr6:105531071-105531130 |
| NM_001005473 | PLCXD3 | -7.719223 | phosphatidylinositol-specific phospholipase C, X domain containing 3 | chr5:41307216-41307157 |
| NM_007037 | ADAMTS8 | -7.720952 | ADAM metallopeptidase with thrombospondin type 1 motif, 8 | chr11:130274879-130274820 |
| NM_001135254 | PAX7 | -7.7515087 | paired box 7 | chr1:19075301-19075360 |
| NM_007129 | ZIC2 | -7.797718 | Zic family member 2 | chr13:100638017-100638076 |
| NM_003106 | SOX2 | -7.83449 | SRY (sex determining region Y)-box 2 | chr3:181431877-181431936 |
| NM_207459 | TEX19 | -7.989044 | testis expressed 19 | chr17:80321545-80321604 |
| NM_006648 | WNK2 | -7.994825 | WNK lysine deficient protein kinase 2 | chr9:96082754-96082813 |
| NM_020134 | DPYSL5 | -8.068495 | dihydropyrimidinase-like 5 | chr2:27172758-27172817 |
| NM_181458 | PAX3 | -8.077288 | paired box 3 | chr2:223064840-223064781 |
| NM_001015001 | CKMT1A | -8.256228 | creatine kinase, mitochondrial 1A | chr15:43988289-43988463 |
| NM_002422 | MMP3 | -8.265078 | matrix metallopeptidase 3 (stromelysin 1, progelatinase) | chr11:102706753-102706694 |
| NM_033176 | NKX2-4 | -8.265844 | NK2 homeobox 4 | chr20:21376070-21376011 |
| NM_006211 | PENK | -8.322184 | proenkephalin | chr8:57354353-57354294 |
| NM_001104587 | SLFN11 | -8.385004 | schlafen family member 11 | chr17:33677430-33677371 |
| NM_198179 | QRFPR | -8.464501 | pyroglutamylated RFamide peptide receptor | chr4:122250849-122250790 |
| NM_000523 | HOXD13 | -8.466869 | homeobox D13 | chr2:176960600-176960659 |
| NM_005222 | DLX6 | -8.743181 | distal-less homeobox 6 | chr7:96640211-96640270 |
